# Identification of early ammonium nitrate-responsive genes in rice roots

**DOI:** 10.1038/s41598-017-17173-9

**Published:** 2017-12-04

**Authors:** Hsiu-Chun Yang, Chia-Cheng Kan, Tzu-Huan Hung, Ping-Han Hsieh, Shi-Yun Wang, Wei-Yu Hsieh, Ming-Hsiun Hsieh

**Affiliations:** 1 Institute of Plant and Microbial Biology, Academia Sinica, Taipei, 11529 Taiwan; 2 Biotechnology Division, Taiwan Agricultural Research Institute, Taichung, 41362 Taiwan

## Abstract

Ammonium has long been used as the predominant form of nitrogen source for paddy rice (*Oryza sativa*). Recently, increasing evidence suggests that nitrate also plays an important role for nitrogen acquisition in the rhizosphere of waterlogged paddy rice. Ammonium and nitrate have a synergistic effect on promoting rice growth. However, the molecular responses induced by simultaneous treatment with ammonium and nitrate have been less studied in rice. Here, we performed transcriptome analysis to identify genes that are rapidly regulated by ammonium nitrate (1.43 mM, 30 min) in rice roots. The combination of ammonium and nitrate preferentially induced the expression of nitrate-responsive genes. Gene ontology enrichment analysis revealed that the early ammonium nitrate-responsive genes were enriched in “regulation of transcription, DNA-dependent” and “protein amino acid phosphorylation” indicating that some of the genes identified in this study may play an important role in nitrogen sensing and signaling. Several defense/stress-responsive genes, including some encoding transcription factors and mitogen-activated protein kinase kinase kinases, were also rapidly induced by ammonium nitrate. These results suggest that nitrogen metabolism, signaling, and defense/stress responses are interconnected. Some of the genes identified here may be involved in the interaction of nitrogen signaling and defense/stress-response pathways in plants.

## Introduction

Nitrogen (N) is one of the most important nutrients for plant growth and development. N deficiency commonly occurs in almost all farmlands, unless N is supplied as a fertilizer or manure. Moreover, the Green Revolution rice cultivars developed in 1960’s (and consequently most of the rice cultivars grown today) require the use of N fertilizers to produce high yields^1^. In the last 50 years, the amounts of synthetic N fertilizers applied to rice have risen dramatically. However, only less than half of the applied N fertilizers can be absorbed by the crop plants^2^, with most of the unused N fertilizer leaching into water or lost to the atmosphere, which can have severe environmental consequences. Application of large amounts of N fertilizers also is economically unsustainable. Therefore, we have an urgent need to develop new solutions to decrease the utilization of N fertilizers while maintaining, or preferably increasing rice production.

N sources in soil or derived from applied N fertilizers are mostly inorganic forms. The inorganic N, mainly nitrate or ammonium, absorbed by plants will be reduced and assimilated into organic N compounds, e.g. glutamine and glutamate, via the glutamine synthetase/glutamine-oxoglutarate aminotransferase (GS/GOGAT) cycle, and these organic forms will be further used for the biosynthesis of other N-containing metabolites to support growth and development. In addition to inorganic N, natural environments also contain amino acids, small polypeptides, and other N-containing organic compounds secreted by living organisms or derived from the decay of organic matter. Plants also can use these organic forms of N^3^. Plants have evolved systems to take up inorganic and organic N to utilize the heterogeneous N sources available in the soil^4^. Still, inorganic N, in particular N fertilizers, provides the predominant N source for crop production. If we can develop a strategy to enhance inorganic N use efficiency in plants, it will not only increase crop yield but also decrease the use of N fertilizers.

Several approaches have manipulated N metabolism and transporter genes to increase N use efficiency in plants. For instance, overexpression of cytosolic GS increased plant height and dry weight under low-N conditions in tobacco^5^. Transgenic maize constitutively overexpressing *GLN1-3*, which encodes a cytosolic GS, in leaves showed a 30% increase in kernel number^6^. Overexpression of NADH-GOGAT in rice produced an increase in grain weight^7^. Overexpression of the *ALANINE AMINOTRANSFERASE* gene also enhanced N use efficiency and biomass in rice and canola^8,9^. Transgenic rice overexpressing an ammonium transporter gene *OsAMT1;1* showed superior growth and higher yield^10^. Overexpression of nitrate transporter genes *OsNRT2.1*, *OsPTR9*, and *OsNRT2.3b*, increased N use efficiency, plant growth, and grain yield in rice^11–13^.

In addition, genetic engineering of N regulatory genes also enabled plants to use N more efficiently. For instance, overexpression of the maize Dof1 transcription factor improved N assimilation and growth under low-N conditions in Arabidopsis and rice^14,15^. Overexpression of *RICE DOF DAILY FLUCTUATION 1* (*RDD1*) increased N responsiveness and grain productivity in rice^16^. Reduced expression of *OsCKX2* encoding a cytokinin oxidase/dehydrogenase caused cytokinin accumulation in inflorescence meristems and increased the number of reproductive organs, resulting in enhanced grain yield in rice^17^.

Transcriptome analysis revealed that the expression of *OsENOD93-1* responded significantly to both increased and decreased N^18^. Interestingly, transgenic rice overexpressing the *OsENOD93-1* gene had increased shoot dry biomass and seed yield^18^. Ectopic expression of a plant-specific gene encoding the G protein γ subunit DEFENSE AND ERECT PANICLES 1 (DEP1) improved harvest index and grain yield at moderate levels of N fertilization^19^. The expression of *DEP1* was positively regulated by N fertilizer^19^.

The identification of N-responsive genes and further manipulation of these genes may enable approaches to produce plants that use N more efficiently. Microarray and RNA-seq analyses have been used to study genome-wide changes in gene expression in response to changes in N conditions. For instance, nitrate-responsive genes have been identified by numerous microarray studies in Arabidopsis and rice^18,20–24^. Transcriptome analyses using microarrays^25^ or RNA-seq.^26^ have identified ammonium-responsive genes in rice. Recently, microarray analysis was used to study responses of rice seedlings to imbalanced carbon/nitrogen availabilities^27^. In addition, microarray and RNA-seq analyses have been applied to identify N starvation responsive genes in rice^28–30^.

Ammonium has long been considered as the primary form of N source for paddy rice due to the anaerobic soil conditions in flooded fields^31^. However, aerenchyma cells in rice roots can transfer oxygen to the rhizosphere, where nitrification of ammonium to nitrate can occur^32^. It has been estimated that 25–40% of total crop N derives from nitrate in the waterlogged paddy rhizosphere^33^. Increased N use efficiency and grain yield in rice overexpressing nitrate transporter genes further confirmed the importance of nitrate for rice^11–13^. As mentioned above, most of the transcriptome analyses used to identify N-responsive genes were conducted with nitrate or ammonium as the sole N source. Here, we examined the effects of ammonium nitrate on the synthesis of amino acids and growth of rice seedlings. Furthermore, microarray analysis was used to identify genes that were rapidly induced by ammonium nitrate in rice roots. Some of the early N-responsive genes identified in this study, especially those encoding transcription factors and protein kinases, may be involved in N sensing and signaling in rice.

## Results

### Effects of ammonium nitrate on the growth of rice seedlings

**Figure 1:**
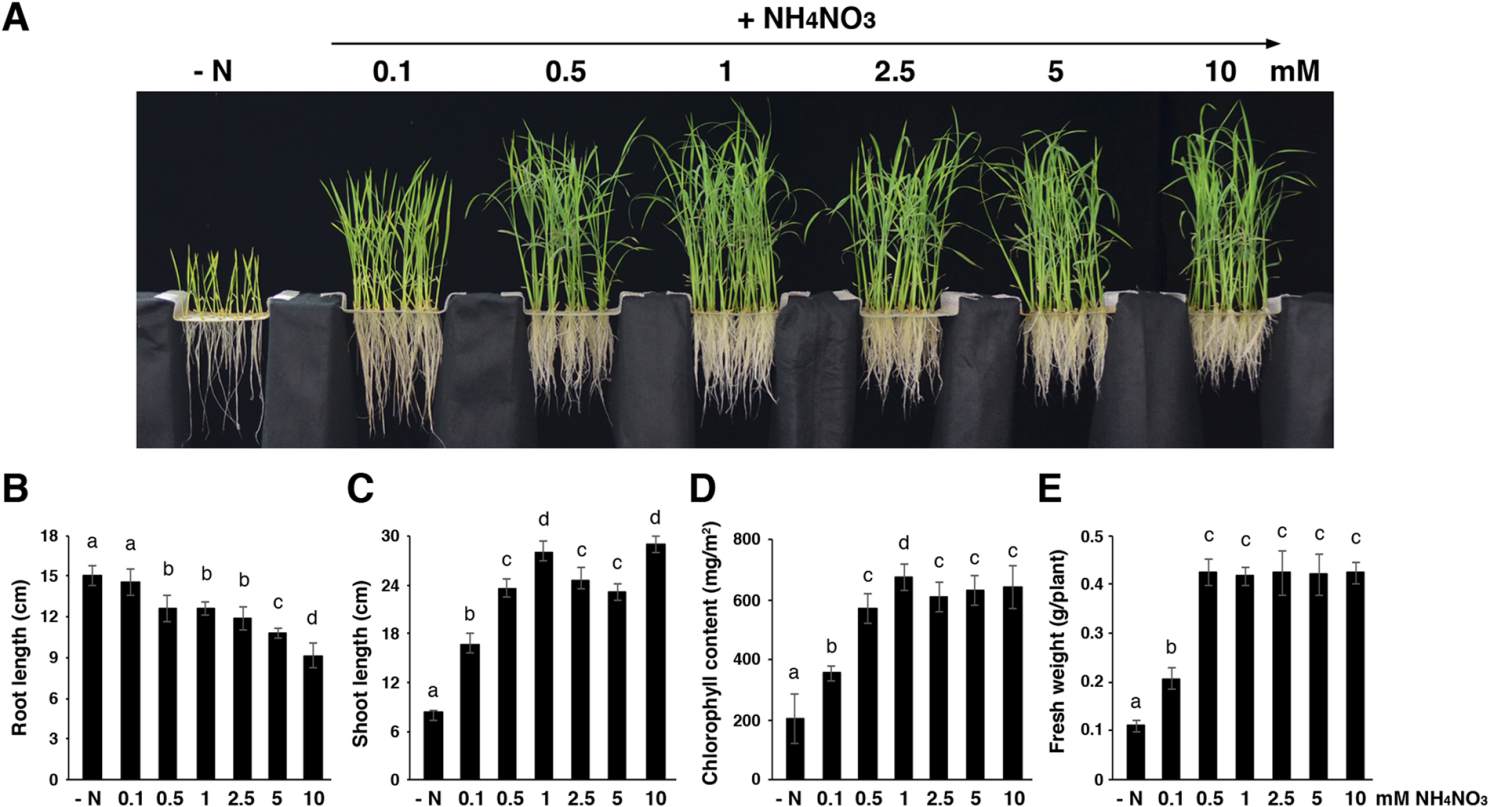
Effects of ammonium nitrate on the growth of rice seedlings. (**A**) Rice seedlings grown in hydroponic solutions containing different concentrations of NH_4_NO_3_ as the nitrogen source. Root length (**B**), shoot length (**C**), chlorophyll contents (**D**), and fresh weight (**E**) of rice seedlings from (**A**). The rice seedlings are 17 days old. Data are means ± SD (*n* = 15). Different letters indicate significant differences between treatments, tested by one-way ANOVA followed by Tukey’s test (*p* < 0.05). −N, no nitrogen.

We used hydroponic solutions to examine the effects of ammonium nitrate on the growth of rice seedlings. The original nitrogen source used by Yoshida *et al*.^34^ was substituted with 0–10 mM ammonium nitrate in the hydroponic solutions. When grown in hydroponics without any nitrogen (-N), the leaves of 17-day-old rice seedlings were chlorotic and the roots were thin and long (Fig. 1A). Supplementation with 0.1 mM ammonium nitrate did not affect the root length (Fig. 1B), but significantly increased the shoot length (Fig. 1C), chlorophyll content (Fig. 1D), and fresh weight of rice seedlings as compared with those grown in -N. Still, rice seedlings grown in 0.1 mM ammonium nitrate had pale green leaves, long and thin roots as compared with those grown in higher concentrations of ammonium nitrate (Fig. 1A). Rice seedlings grown in 0.5–10 mM ammonium nitrate developed more crown and lateral roots, but their roots were shorter than those grown in -N or 0.1 mM ammonium nitrate (Fig. 1A,B). In addition, rice seedlings grown in 0.5–10 mM ammonium nitrate had similar fresh weight, which was significantly higher than those grown in -N or 0.1 mM ammonium nitrate (Fig. 1E). The results for shoot length, chlorophyll content, and fresh weight suggested that the optimal concentration of ammonium nitrate for growing rice seedlings in the hydroponics was 1–2.5 mM. Therefore, we used the original concentration of ammonium nitrate, e.g. 1.43 mM, recommended by Yoshida *et al*.^34^ in the following experiments.

### Effects of ammonium nitrate on the endogenous levels of amino acids

**Figure 2:**
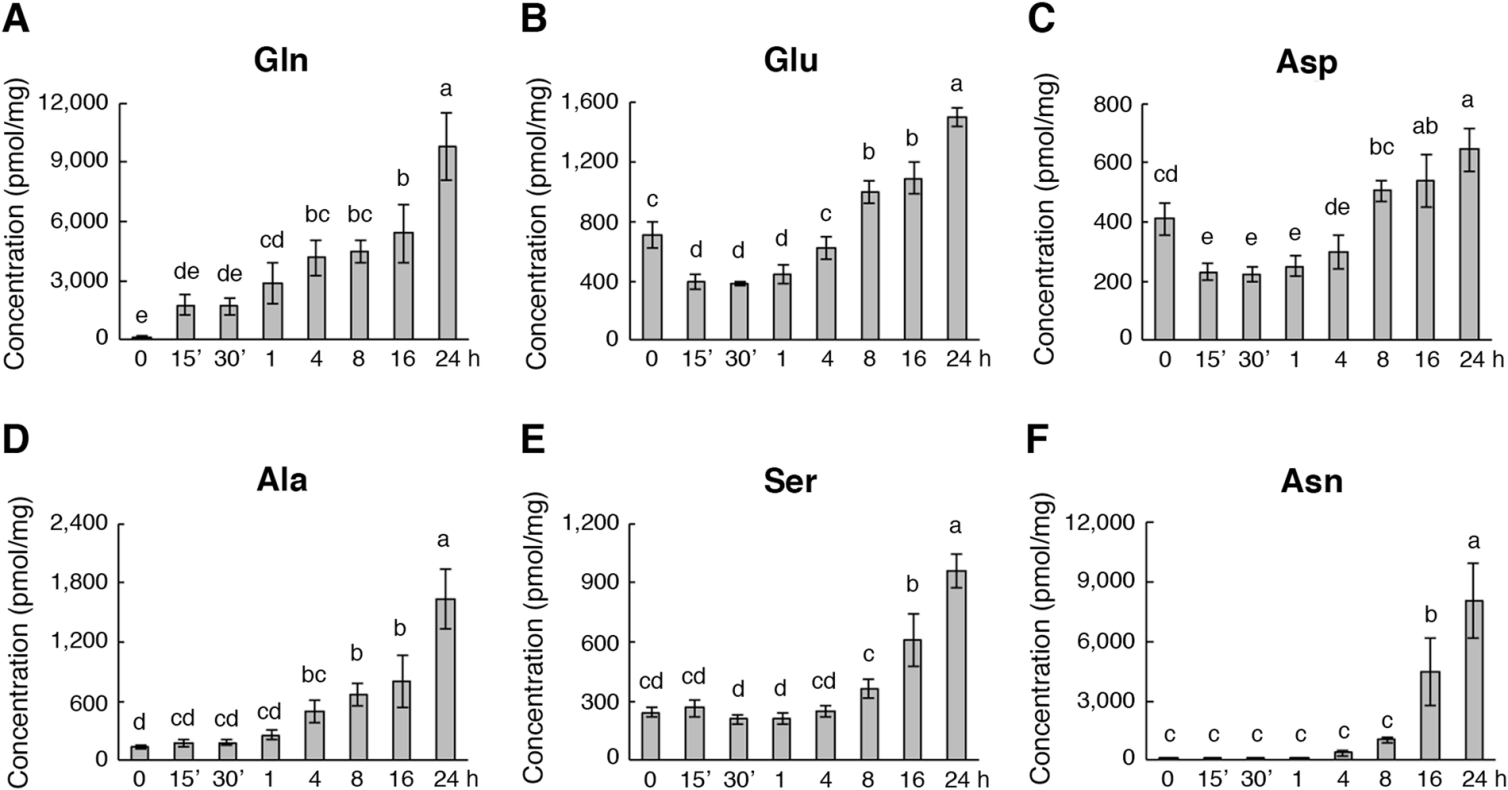
Amino acid contents in the rice roots during the time course of ammonium nitrate treatment. (**A**–**F**) Contents of glutamine, glutamate, aspartate, alanine, serine, and asparagine in the roots of 17-day-old nitrogen-starved rice seedlings after 0–24 h of 1.43 mM ammonium nitrate treatment. Data are means ± SD (*n* = 4). Different letters indicate significant differences between treatments, tested by one-way ANOVA followed by Tukey’s test (*p* < 0.05).

Ammonium nitrate taken up by roots will be assimilated into organic N such as amino acids to support plant metabolism, growth, and development. We examined the effects of ammonium nitrate on the endogenous levels of amino acids in rice roots, and the results for the six most abundant amino acids, glutamine (Gln), glutamate (Glu), aspartate (Asp), alanine (Ala), serine (Ser), and asparagine (Asn), are shown in Fig. 2. Glu was the most abundant amino acid in the roots of N-starved rice seedlings^35,36^. Feeding of ammonium nitrate to N-starved rice seedlings rapidly increased the endogenous levels of Gln within 15–30 min, but not Glu or the other amino acids (Fig. 2, Supplementary Fig. S1). Levels of Gln continued to increase after 1–24 h of ammonium nitrate treatment (Fig. 2A). By contrast, the content of free Glu decreased within the first hour and increased significantly after 8–24 h in the roots of ammonium nitrate-treated rice seedlings (Fig. 2B). During the time course of ammonium nitrate treatment, the accumulation of free Asp displayed a pattern similar to that of Glu (Fig. 2C). The content of Ala started to increase after 4 h (Fig. 2D), and levels of Ser and Asn increased significantly after 16 h of ammonium nitrate treatment (Fig. 2E,F). The effects of ammonium nitrate on the accumulation of the other amino acids were shown in Supplementary Fig. S1. The amounts of phenylalanine, lysine, valine, arginine, glycine, and threonine increased after 16–24 h, and the levels of histidine decreased slightly after 4–24 h of ammonium nitrate treatment (Supplementary Fig. S1A). By contrast, the levels of cysteine, threonine, tryptophan, isoleucine, leucine, proline, and methionine in rice roots were not significantly affected by the ammonium nitrate treatment (Supplementary Fig. S1B). These results indicated that Gln is the only amino acid that increased significantly within 15–30 min of ammonium nitrate treatment in N-starved rice roots.

### Identification of early ammonium nitrate-responsive genes

We used microarray analysis to identify differentially expressed genes (DEGs) in N-starved (−N) and ammonium nitrate-treated (1.43 mM, 30 min; +N) rice roots. The results revealed that 196 genes, including 189 up- and 7 down-regulated, were differentially expressed with 2-fold cutoff (+N/−N). We further used quantitative (q) RT-PCR analysis to examine the effects of the ammonium nitrate time course (0–24 h) treatment on the expression of all 196 genes, and were able to confirm that the expression of 162 genes (158 up and 4 down) was differentially regulated by +N (see below). A list of the 162 DEGs is shown in Supplementary Table S1.

Feeding of ammonium nitrate to N-starved rice seedlings is expected to rapidly induce the expression of genes involved in N uptake and assimilation. Kyoto Encyclopedia of Genes and Genomes (KEGG) enrichment analysis of the 158 up-regulated genes revealed that “photosynthesis (ko00195)”, “nitrogen metabolism (ko00910)”, “sulfur metabolism (ko00920)”, “carbon metabolism (ko01200)”, “pentose phosphate pathway (ko00030)”, “glutathione metabolism (ko00480)”, and “C5-branched dibasic acid metabolism (ko00660)” were enriched. A list of genes enriched in these pathways is shown in Supplementary Table S2.

### Ammonium nitrate preferentially induced the expression of genes involved in nitrate uptake and assimilation

**Figure 3:**
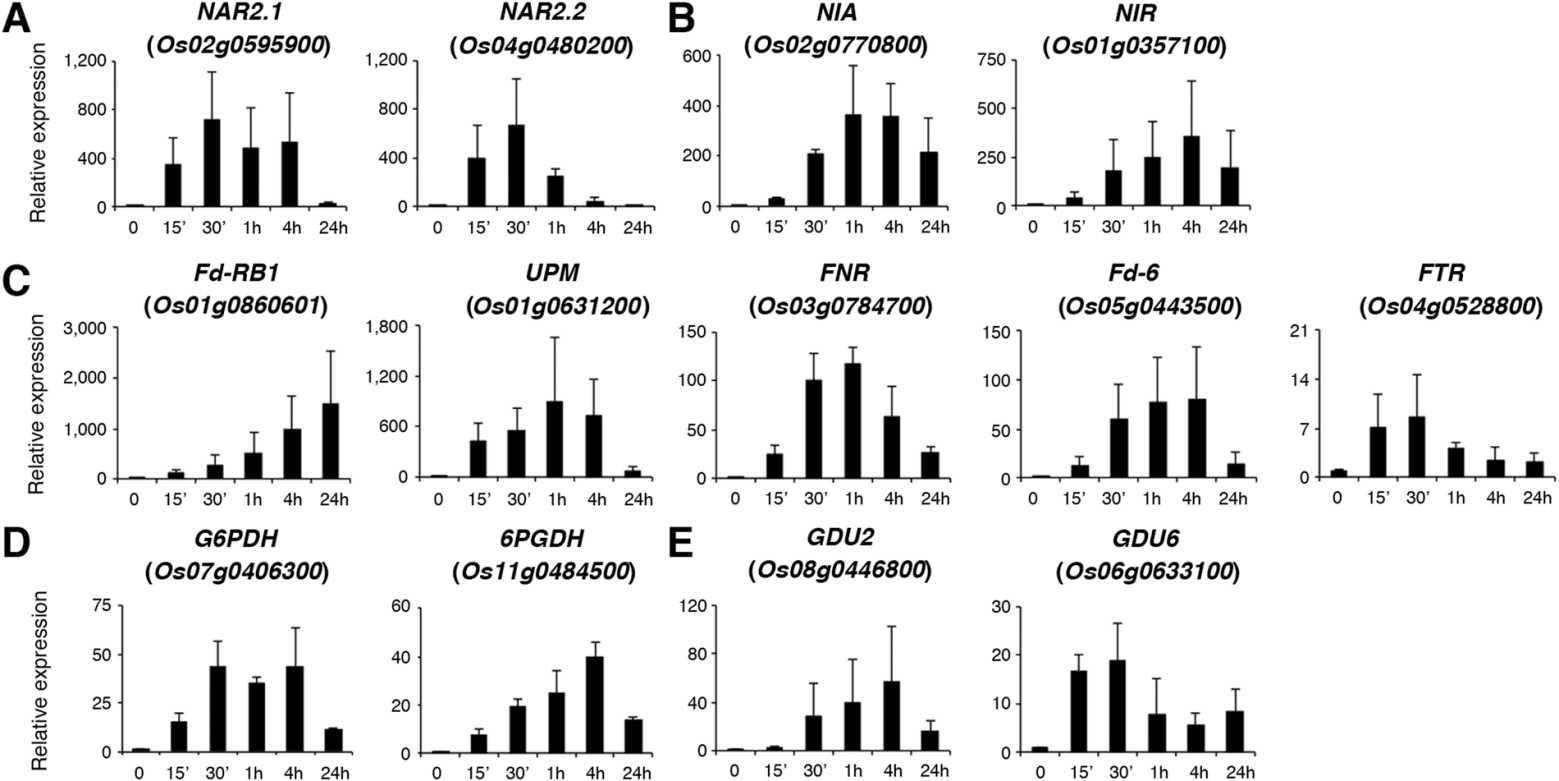
Quantitative RT-PCR analysis of ammonium nitrate-responsive genes. Total RNA extracted from roots of 17-day-old rice seedlings treated with ammonium nitrate for 0–24 h was used for qRT-PCR analysis to verify the expression of genes involved in nitrate transport (**A**), nitrate/nitrite assimilation (**B**), ferredoxin reduction (**C**), the pentose phosphate pathway (**D**), and amino acid transport (**E**). Relative expression indicates the fold-change of each gene as compared to that of control. Results are shown as means ± SD from three biological repeats.

It is well known that nitrate can induce the expression of genes involved in nitrate uptake, nitrate/nitrite assimilation, ferredoxin reduction, and the pentose phosphate pathway^21^. We found that the expression of these nitrate-responsive genes was also rapidly induced by ammonium nitrate in rice roots (Table 1). These results suggest that simultaneous treatment with ammonium and nitrate may preferentially induce the expression of genes involved in nitrate uptake and assimilation. In addition to the above well-known nitrate-responsive genes, the expression of two *GLUTAMINE DUMPER* genes was also rapidly induced by ammonium nitrate (Table 1). We used qRT-PCR analysis to examine the expression of genes listed in Table 1 during the time course of ammonium nitrate treatment (1.43 mM, 0–24 h) in rice roots. The expression of two nitrate transporter genes, *NAR2.1* and *NAR2.2*, was rapidly and strongly induced by ammonium nitrate (Fig. 3A). Similarly, the expression of *NITRATE REDUCTASE* (*NIA*, *Os02g0770800*) and *NITRITE REDUCTASE* (*NIR*, *Os01g0357100*) was highly induced during the time course of ammonium nitrate treatment (Fig. 3B). Ferredoxin (Fd), uroporphyrinogen-III C-methyltransferase (UPM), Fd-NADH reductase (FNR), and Fd-thioredoxin reductase (FTR) are indirectly involved in nitrite reduction. The pentose phosphate pathway enzymes glucose-6-phosphate 1-dehydrogenase (G6PDH) and 6-phosphogluconate dehydrogenase (6PGDH) are directly involved in the production of NADPH, which is required for nitrate reduction. The expression of the genes encoding these enzymes was rapidly induced by ammonium nitrate (Fig. 3C,D). In addition, the expression of two *GLUTAMINE DUMPER* genes, *GDU2* (*Os08g0446800*) and *GDU6* (*Os06g0633100*), was also induced during the time course of ammonium nitrate treatment (Fig. 3E).

**Table 1:** Selected genes rapidly up-regulated by ammonium nitrate in rice roots.

Gene Identifier	*Fold change	Gene description
Nitrate uptake		
Os04g0480200 LOC_Os04g40410	7.0	High-affinity nitrate transporter 2.2, NAR2.2
Os02g0595900 LOC_Os02g38230	5.2	High-affinity nitrate transporter 2.1, NAR2.1
Nitrate/nitrite assimilation		
Os02g0770800 LOC_Os02g53130	65.8	Nitrate reductase [NAD(P)H], NIA
Os01g0357100 LOC_Os01g25484	8.0	Ferredoxin–nitrite reductase, NIR
Ferredoxin reduction		
Os01g0860601 LOC_Os01g64120	42.2	Ferredoxin, root R-B1
Os01g0631200 LOC_Os01g44050	32.3	Uroporphyrinogen-III C-methyltransferase, UPM
Os03g0784700 LOC_Os03g57120	8.5	Ferredoxin–NADP reductase, FNR
Os05g0443500 LOC_Os05g37140	2.9	Ferredoxin-6
Os04g0528800 LOC_Os04g44650	2.1	Ferredoxin-thioredoxin reductase, FTR
Pentose phosphate pathway		
Os07g0406300 LOC_Os07g22350	5.1	Glucose-6-phosphate 1-dehydrogenase, G6PDH
Os11g0484500 LOC_Os11g29400	4.3	6-Phosphogluconate dehydrogenase, 6PGDH
Amino acid transport		
Os06g0633100 LOC_Os06g42660	4.9	Glutamine dumper 6, GDU6
Os08g0446800 LOC_Os08g34700	2.1	Glutamine dumper 2, GDU2

Total RNA extracted from roots of 17-day-old rice seedlings grown in hydroponic solution without nitrogen (−N) or treated with 1.43 mM ammonium nitrate for 30 min (+N) was used for microarray analysis. *Fold change indicates the ratio of +N/−N.

### Ammonium nitrate rapidly induced the expression of genes encoding transcription factors and protein kinases/phosphatases

**Figure 4:**
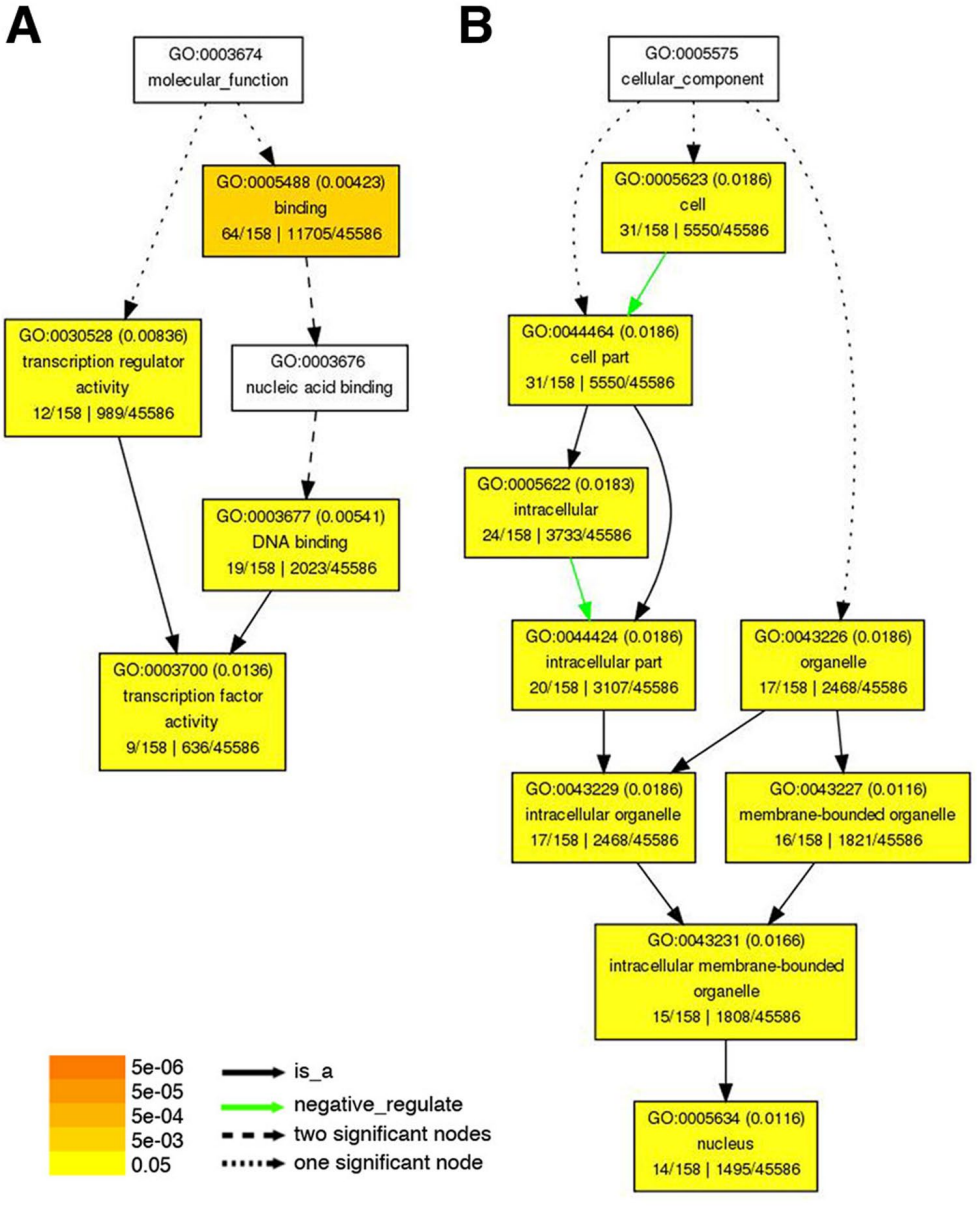
Gene ontology enrichment analysis of genes up-regulated by ammonium nitrate. The differentially expressed genes were analyzed by enrichment analysis using AgriGO. Significantly enriched GO categories in molecular function (**A**) and cellular component (**B**) are shown in yellow and orange (false discovery rate, FDR < 0.05).

Gene Ontology (GO) analysis provides defined terms that represent gene product properties in three domains: molecular function, cellular component, and biological process (http://www.geneontology.org). In addition to KEGG pathway enrichment analysis, we used agriGO (http://bioinfo.cau.edu.cn/agriGO/) to perform Gene Ontology (GO) enrichment analysis of the 158 up-regulated genes. The results of this analysis show which GO terms are over-represented in the ammonium nitrate-induced genes. Of several visualization methods provided by the agriGO software, we selected the direct acyclic graph (DAG) tree to present the results (Fig. 4 and Supplementary Fig. S2). In the DAG tree, GO terms are represented as boxes containing a detailed description; the boxes are organized and connected based on their relationship. In molecular function, the GO term “transcription factor activity” was significantly enriched (Fig. 4A). In cellular component, the GO term “nucleus” was significantly enriched (Fig. 4B). In biological process, the GO terms “regulation of transcription, DNA-dependent”, “protein amino acid phosphorylation”, and “signal transduction” were significantly enriched (Supplementary Fig. S2). These results indicated that treatment of N-starved rice seedlings with 1.43 mM ammonium nitrate for 30 min preferentially induced the expression of transcription factor/nuclear protein and protein kinase genes. These transcription factors/nuclear proteins and protein kinases may amplify and/or mediate the signals derived from ammonium nitrate to regulate the downstream molecular and cellular responses in rice roots. A list of genes derived from the GO enrichment analysis is shown in Supplementary Table S3.

In addition to GO analysis, we went through the annotation and literature for the 158 up-regulated genes in the National Center for Biotechnology Information (NCBI, https://www.ncbi.nlm.nih.gov) and found at least 35 genes encoding putative transcription factors or nuclear proteins, 12 genes encoding protein kinases, and 2 genes encoding phosphatases (Table 2). Arabidopsis LBD/37/38/39 transcription factors and calcineurin B-like protein (CBL)-interacting protein kinases (CIPKs) are involved in N sensing and signaling^37–39^. Interestingly, several rice genes encoding homologs of LBD/37/38/39 and CIPKs were identified in this study (Table 2).

**Table 2:** Early ammonium nitrate-responsive genes encoding transcription factors/nuclear proteins in rice roots.

No.	Locus ID		Fold change*	Gene description
**Transcription factor/nuclear protein**				
1	Os03g0609500	LOC_Os03g41330	30.5	LOB domain-containing protein 38 (LBD38)
2	Os02g0728001	LOC_Os02g49560	17.2	Basic leucine zipper 43-like (bZIP 43-like)
3	Os03g0445700	LOC_Os03g33090	15.0	LOB domain-containing protein 37 (LBD37)
4	Os05g0525900	LOC_Os05g45020	14.7	Zinc finger CCCH domain-containing protein 37
5	Os09g0522200	LOC_Os09g35030	14.7	Dehydration-responsive element-binding protein 1 A (DREB1A)
6	Os05g0114400	LOC_Os05g02390	14.0	Zinc finger protein, ZOS5-02
7	Os11g0184900	LOC_Os11g08210	11.5	NAC domain-containing protein, NAC5
8	Os07g0119300	LOC_Os07g02800	9.4	Unknown, MYB family protein
9	Os07g0589000	LOC_Os07g40000	8.5	LOB domain-containing protein 37 (LBD37)
10	Os03g0764600	LOC_Os03g55590	8.0	Unknown, MYB family protein
11	Os01g0130900	LOC_Os01g03980	6.2	Zinc finger protein ZIC 2
12	Os01g0752500	LOC_Os01g54890	5.0	PTI5; AP2/ERF transcription factor
13	Os01g0733200	LOC_Os01g53220	4.7	Heat stress transcription factor C-1b
14	Os01g0780800	LOC_Os01g57240	4.1	ULTRAPETALA 1 (ULT1), trithorax group factor
15	Os07g0583600	LOC_Os07g39470	3.8	CIGR2; GRAS family protein
16	Os02g0713700	LOC_Os02g48320	3.6	AT-hook motif nuclear-localized protein 25
17	Os01g0948200	LOC_Os01g71970	3.6	Scarecrow-like protein 3; GRAS family protein
18	Os02g0775600	LOC_Os02g53530	3.5	Zinc finger protein 8
19	Os02g0158000	LOC_Os02g06330	3.0	AP2/ERF transcription factor
20	Os04g0567800	LOC_Os04g47990	2.8	Dof zinc finger protein DOF4.6
21	Os06g0166400	LOC_Os06g07030	2.8	Ethylene-responsive transcription factor RAP2-9
22	Os07g0593000	LOC_Os07g40300	2.7	Zinc finger protein 7 (ZFP7)
23	Os04g0450900	LOC_Os04g37790	2.7	SMC chromosome segregation protein
24	Os02g0530300	LOC_Os02g32840	2.6	Zinc finger A20, stress-associated protein 5 (SAP5)
25	Os05g0560200	LOC_Os05g48650	2.5	Seed dormancy control (DOG1), transcription factor-like
26	Os03g0230300	LOC_Os03g12820	2.5	Similar to RCD One protein 2 (SRO2)
27	Os01g0797600	LOC_Os01g58420	2.4	Ethylene-responsive transcription factor 8
28	Os01g0908200	LOC_Os01g68020	2.4	BTB/POZ and TAZ domain-containing protein 2
29	Os07g0602900	LOC_Os07g41160	2.3	Ninja-family protein
30	Os07g0685700	LOC_Os07g48630	2.1	ETHYLENE INSENSITIVE 3-like 1 protein, EIL2
31	Os07g0225300	LOC_Os07g12340	2.1	NAC domain-containing protein 67
32	Os04g0460600	LOC_Os04g38720	2.1	NAC domain-containing protein 92
33	Os09g0522000	LOC_Os09g35010	2.1	Dehydration-responsive element-binding protein 1B (DREB1B)
34	Os04g0648900	LOC_Os04g55520	2.1	Ethylene-responsive transcription factor ERF008
35	Os06g0107800	LOC_Os06g01860	2.0	B3 DNA binding domain-containing protein
**Protein kinase/phosphatase**				
1	Os01g0699100	LOC_Os01g50370	8.2	Mitogen-activated protein kinase kinase kinase 63 (MAPKKK63)
2	Os11g0113700/ Os12g0113500	LOC_Os11g02240/ LOC_Os12g02200	5.4	CBL-interacting protein kinase 15 (CIPK15) CBL-interacting protein kinase 14 (CIPK14)
3	Os01g0699600	LOC_Os01g50420	3.6	Mitogen-activated protein kinase kinase kinase 62 (MAPKKK62)
4	Os02g0623600	LOC_Os02g41480	3.5	Wall-associated receptor kinase 5 (WAK5)
5	Os09g0418000	LOC_Os09g25090	3.5	CBL-interacting protein kinase 16 (CIPK16)
6	Os01g0699400	LOC_Os01g50400	2.9	Mitogen-activated protein kinase kinase kinase 55 (MAPKKK55)
7	Os07g0584100	LOC_Os07g39520	2.9	Mitogen-activated protein kinase kinase kinase 64 (MAPKKK64)
8	Os05g0545400	LOC_Os05g46760	2.7	Mitogen-activated protein kinase kinase kinase 69 (MAPKKK69)
9	Os01g0292200	LOC_Os01g18800	2.7	CBL-interacting protein kinase 1 (CIPK1)
10	Os01g0699500	LOC_Os01g50410	2.2	Mitogen-activated protein kinase kinase kinase 70 (MAPKKK70)
11	Os07g0538400	LOC_Os07g35390	2.2	Cysteine-rich receptor-like protein kinase 25 (CRK25)
12	Os02g0767400	LOC_Os02g52850	2.1	G-type lectin S-receptor-like Ser/Thr-protein kinase (SRK)
13	Os06g0208700	LOC_Os06g10650	2.5	Plant and fungi atypical dual-specificity phosphatase (PFA-DSP)
14	Os10g0541200	LOC_Os10g39540	2.2	Phosphatase 2 C 47

Total RNA extracted from roots of 17-day-old rice seedlings grown in hydroponic solution without nitrogen (-N) or treated with 1.43 mM ammonium nitrate for 30 min ( + N) was used for microarray analysis. *Fold change indicates the ratio of + N/-N.

**Figure 5:**
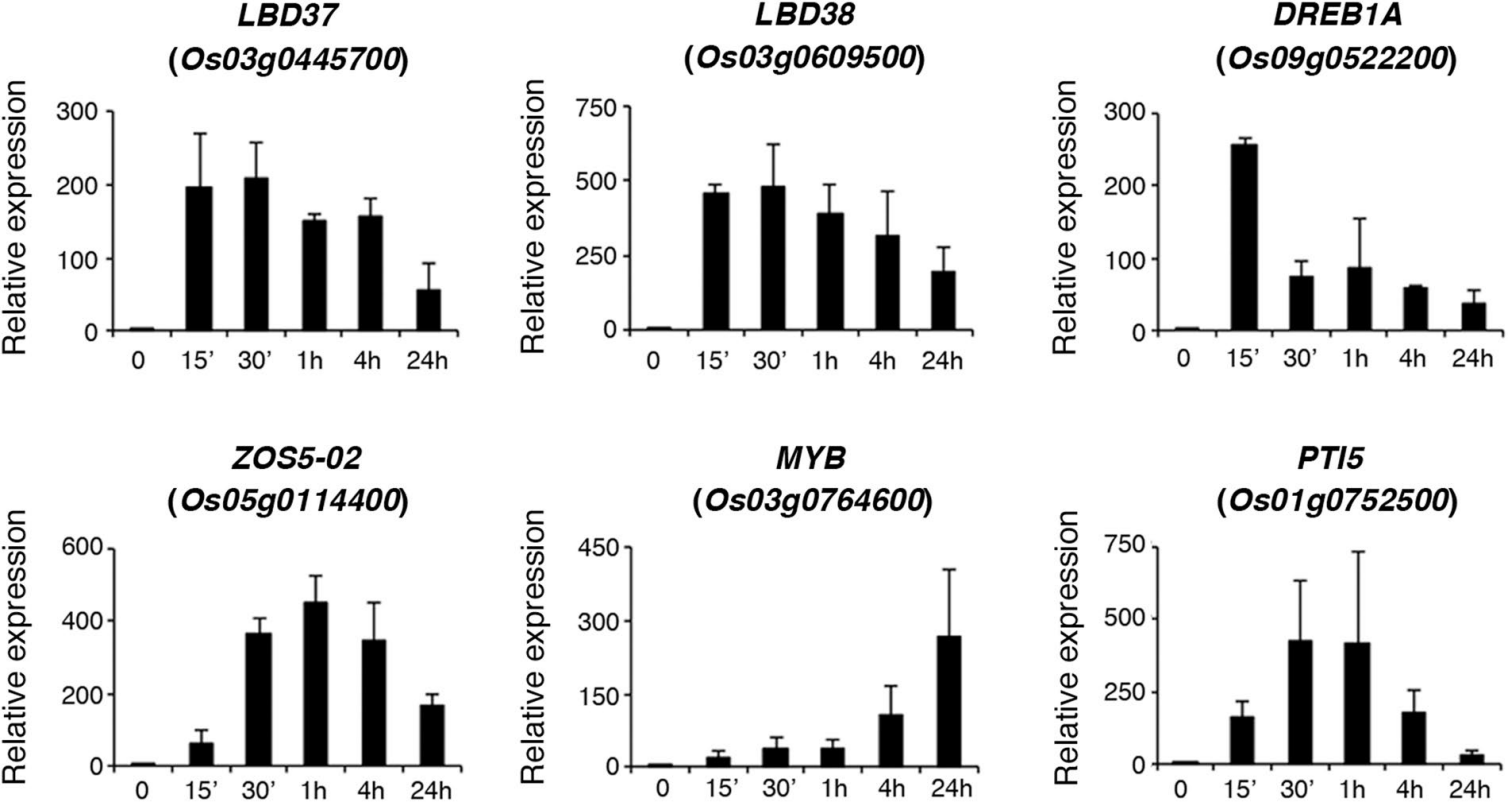
Quantitative RT-PCR analysis of representative transcription factor genes that are rapidly and strongly induced by ammonium nitrate. Total RNA extracted from roots of 17-day-old rice seedlings treated with ammonium nitrate for 0–24 h was used for qRT-PCR to analyze the expression of transcription factor genes *LBD37*, *LBD38*, *DREB1A*, *ZOS5–02*, *MYB*, and *PTI5*. Relative expression indicates the fold-change of each gene as compared to that of control. Results are shown as means ± SD from three biological repeats.

We also used qRT-PCR analysis to verify that the expression of these 49 genes was rapidly induced by ammonium nitrate. Of the 35 genes encoding putative transcription factors or nuclear proteins, the expression of the following 6 transcription factor genes was rapidly (15 min–4 h) and strongly (>100-fold) induced by ammonium nitrate: *LBD37* (*Os03g0445700*, *LOC_Os03g33090*) encoding LOB domain-containing protein 37, *LBD38* (*Os03g0609500*, *LOC_Os03g41330*) encoding LOB domain-containing protein 38, *DREB1A* (*Os09g0522200*, *LOC_Os09g35030*) encoding dehydration-responsive element-binding protein 1 A, *ZOS5–02* (*Os05g0114400*, *LOC_Os05g02390*) encoding a zinc finger protein, *MYB (Os03g0764600*, *LOC_Os03g55590*) encoding a MYB family protein, and *PTI5* (*Os01g0752500*, *LOC_Os01g54890*) encoding pathogenesis-related genes transcriptional activator 5, an AP2/ERF transcription factor (Fig. 5). The expression patterns of the other 29 genes encoding putative transcription factors or nuclear proteins during the time course of ammonium nitrate treatment are shown in Supplementary Fig. S3.

**Figure 6:**
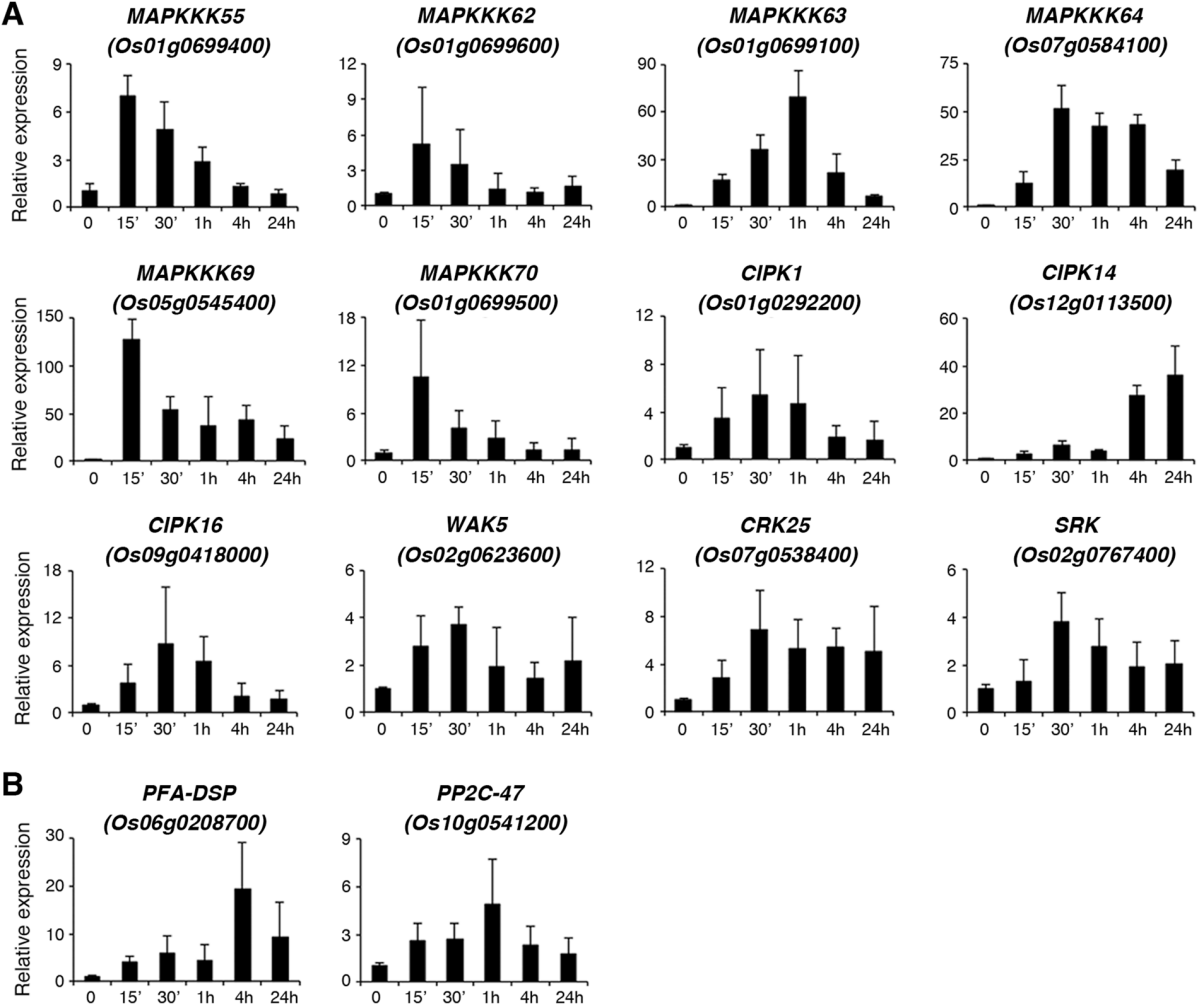
Regulation of nitrogen-responsive protein kinase/phosphatase genes by ammonium nitrate. Total RNA extracted from roots of 17-day-old rice seedlings treated with ammonium nitrate for 0–24 h was used for qRT-PCR to analyze the expression of protein kinase (**A**) and phosphatase (**B**) genes. Relative expression indicates the fold-change of each gene as compared to that of control. Results are shown as means ± SD from three biological repeats.

The effects of the ammonium nitrate time course on the expression of 12 protein kinase genes, including 6 genes encoding mitogen-activated protein kinase kinase kinases (MAPKKKs), 3 genes encoding CIPKs, 2 genes encoding receptor-like protein kinases, and one gene encoding a wall-associated receptor kinase (WAK), and two genes encoding protein phosphatases, are shown in Fig. 6A,B. Besides the genes shown in Figs 3, 5, 6 and Supplementary Fig. S3, the expression patterns of the other 96 genes during the time course of ammonium nitrate treatment are shown in Supplementary Fig. S4.

### Analysis of ammonium nitrate repressed genes

**Figure 7:**
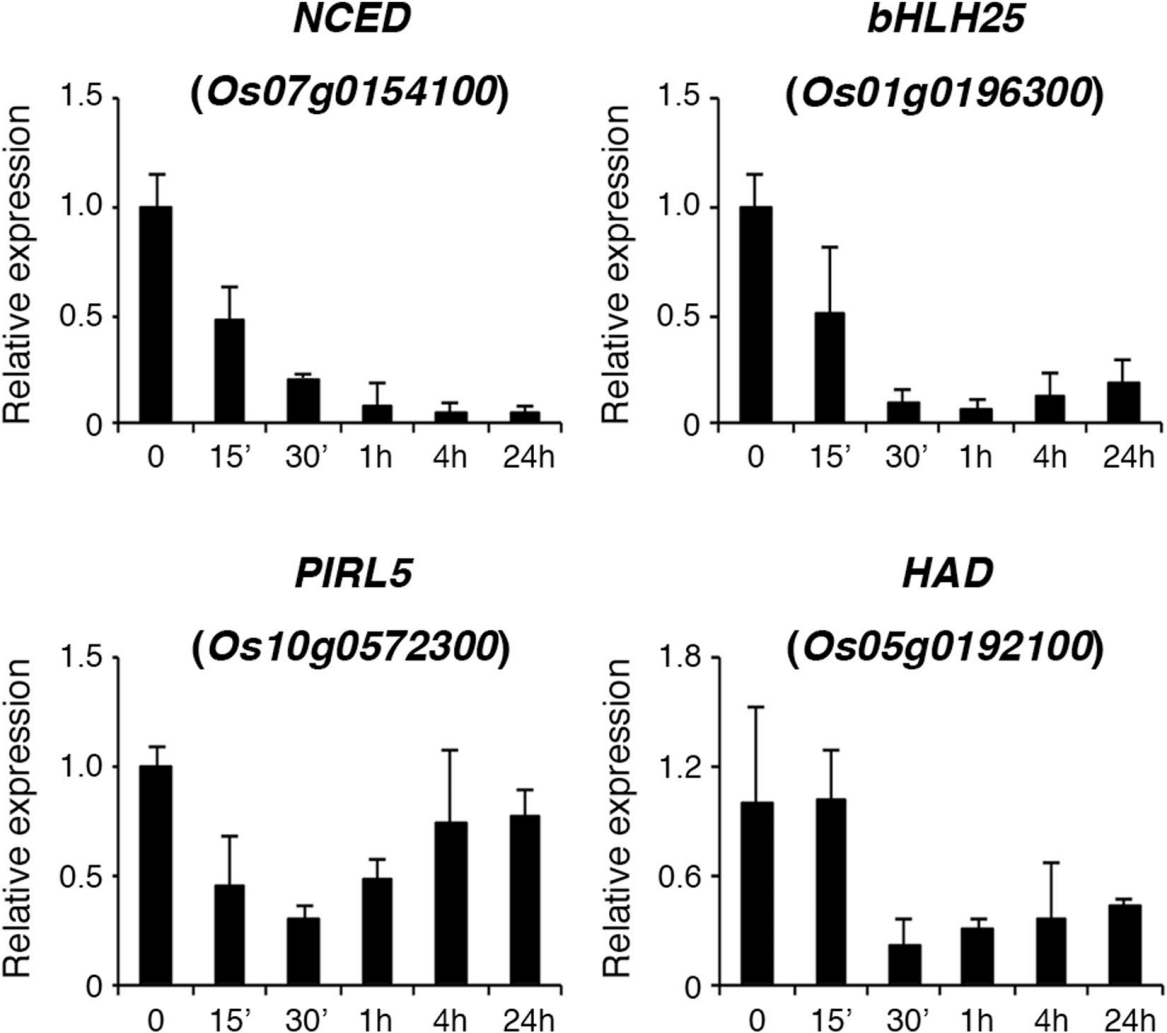
Ammonium nitrate rapidly repressed the expression of *NCED, bHLH25, PIRL5*, and *HAD* genes. Seventeen-day-old nitrogen-starved rice seedlings were transferred to hydroponics containing 1.43 mM ammonium nitrate for 0–24 h. Total RNA extracted from roots was used for qRT-PCR analysis. Relative expression indicates the fold-change of each gene as compared to that of control. Results are shown as means ± SD from three biological repeats.

Only four genes were rapidly repressed by ammonium nitrate in rice roots. Among these genes, the expression of *Os07g0154100*, encoding 9-cis-epoxycarotenoid dioxygenase (NCED), a key enzyme in abscisic acid (ABA) biosynthesis, was rapidly and strongly repressed by ammonium nitrate (Fig. 7). These results suggest that treatment of N-starved rice seedlings with ammonium nitrate may inhibit ABA biosynthesis and signaling in the roots. The expression of the other three genes, including *Os01g0196300* encoding basic helix-loop-helix domain-containing protein 25 (bHLH25), *Os10g0572300* encoding the intracellular Ras-group-related LRR protein 5 (IRL5), and *Os05g0192100* encoding a haloacid dehydrogenase (HAD) domain-containing protein, during the time course of ammonium nitrate treatment are shown in Fig. 7.

### Induction of early ammonium nitrate-responsive genes in the shoots

**Figure 8:**
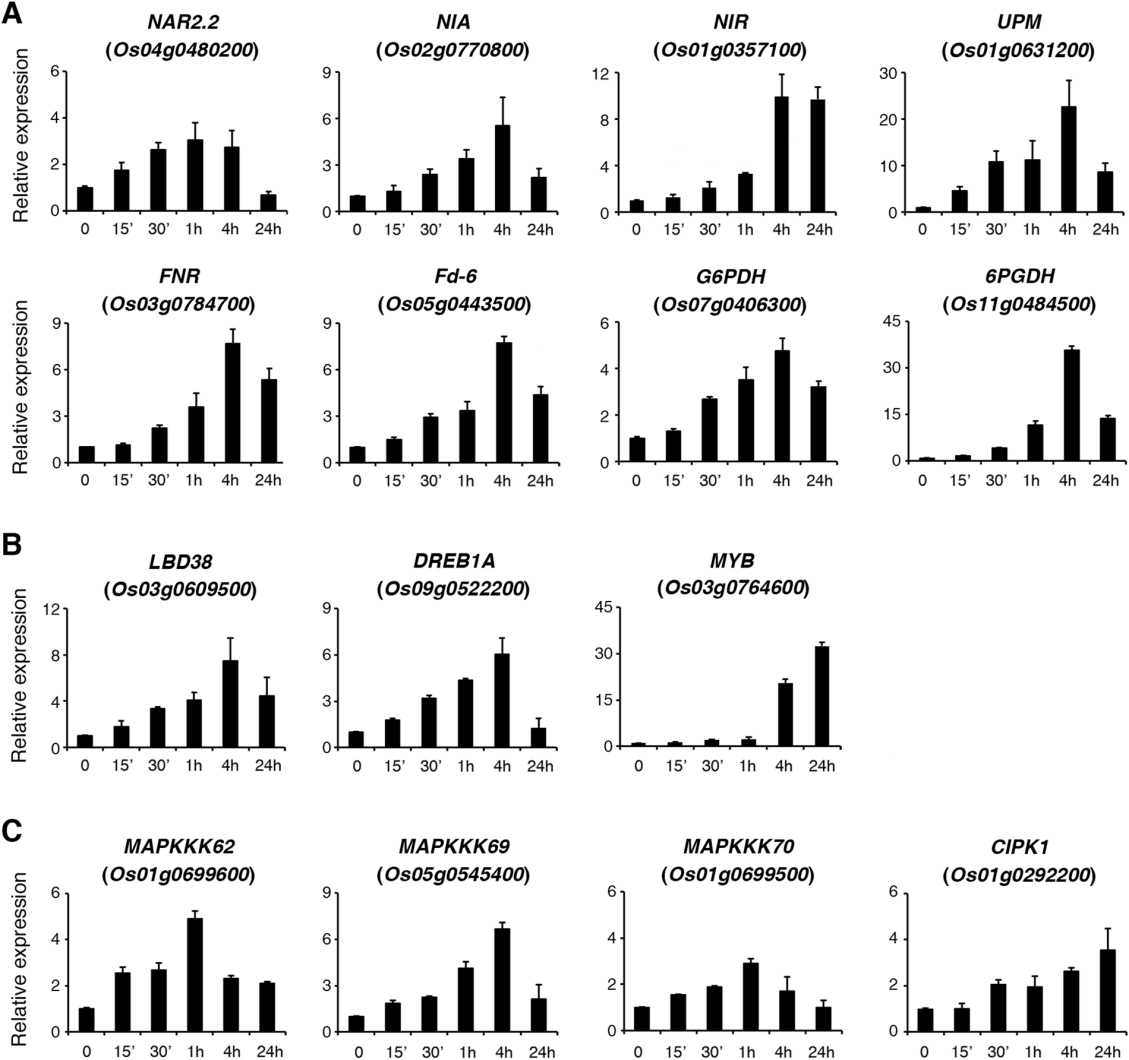
Quantitative RT-PCR analysis of genes induced by ammonium nitrate in the shoots. Seventeen-day-old nitrogen-starved rice seedlings were transferred to hydroponic solution containing 1.43 mM ammonium nitrate for 0–24 h. Total RNA extracted from shoots was used for qRT-PCR analysis. Relative expression indicates the fold-change of each gene as compared to that of control. Results are means ± SD from three biological repeats.

To test if the early ammonium nitrate-responsive genes identified in the roots were also induced by the same treatment in the shoots, we used qRT-PCR analysis to measure the expression of selected genes during a time course of ammonium nitrate treatment (1.43 mM, 0–24 h). The genes examined include nitrate-responsive genes, transcription factor genes and protein kinase/phosphatase genes shown in Figs 3, 5 and 6, respectively. The results revealed that most of the well known nitrate-responsive genes induced by ammonium nitrate in the roots were also rapidly induced by ammonium nitrate in the shoots (Fig. 8A). However, the induction of these genes in the shoots was not as strong as that observed in the roots. For instance, the induction of *NAR2.2*, *NIA*, and *NIR* was only 2–3 fold in the shoots (Fig. 8A), whereas the induction of these genes was more than 200 fold in the roots (Fig. 3) after treatments with ammonium nitrate for 0.5–1 h. In addition, the expression of nitrate-responsive genes in the shoots seemed to peak at a later time point, as compared with the expression in the roots during the time course of ammonium nitrate treatment (compare Figs 3 and 8A). The expression of *Fd-RB1* and *FTR* in the shoots was not significantly induced by ammonium nitrate until treatment had occurred for 4–24 h (Supplemental Fig. S5A). While the expression of *GDU2* and *GDU6* was rapidly and strongly induced by ammonium nitrate in the roots, the expression of these gene was not significantly induced in the shoots under the same treatments (Supplemental Fig. S5A).

In contrast to the well known nitrate-responsive genes, many of the transcription factor and protein kinase/phosphatase genes identified in this study seemed to be specifically induced by ammonium nitrate in the roots. Of the 20 transcription factor and protein kinase/phosphatase genes examined here (Figs 5 and 6), only 7 genes were rapidly and significantly (>2 fold, 0.5–1 h treatment) induced by ammonium nitrate in the shoots (Fig. 8B,C). Similar to the trends observed in the nitrate-responsive genes, the expression of the transcription factor and protein kinase genes in the shoots peaked at a later time point, and the induction of these genes was weaker than that observed in the root samples during the time course of ammonium nitrate treatment (compare Figs 5 and 6 with Fig. 8B,C). The expression of the other 13 genes, except *LBD37* and *CIPK14*, was not induced or only slightly induced in the shoots during the time course of ammonium nitrate treatment (Supplemental Fig. S5B,C). The expression of *LBD37* and *CIPK14* in the shoots was induced approximately 5–6 fold after 24 h of ammonium nitrate treatment (Supplemental Fig. S5B,C). These results suggest that many of the early ammonium nitrate-responsive genes, especially transcription factor and protein kinase genes, identified in this study are specific to roots. Genes commonly induced by ammonium nitrate in both roots and shoots are expected to have a slower and weaker response in the shoots, as the treatment was conducted on the roots of rice seedlings grown in hydroponic solution.

### Genes commonly induced by ammonium nitrate, glutamine, and glutamate

**Figure 9:**
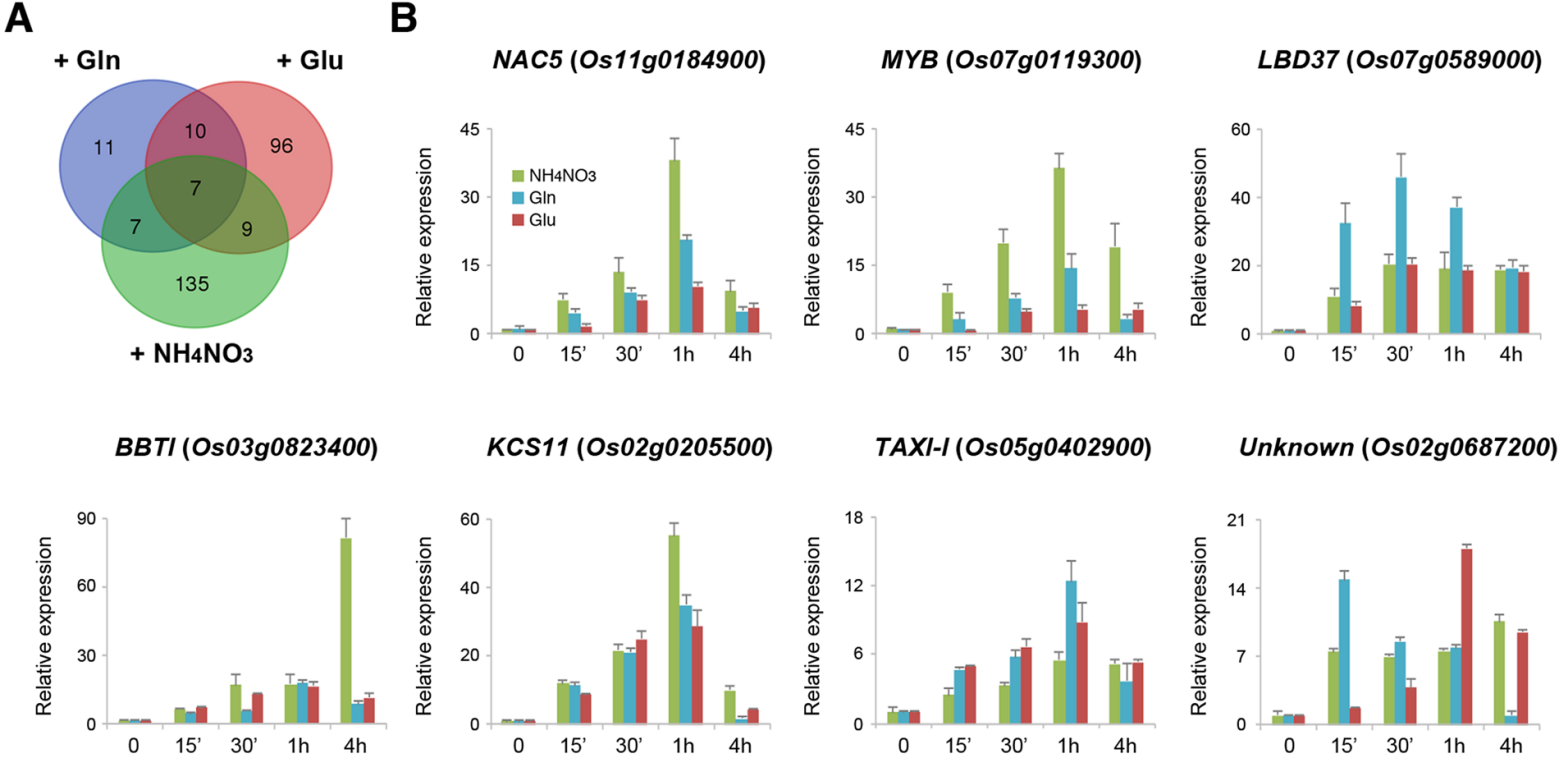
Identification of genes commonly induced by glutamine, glutamate, and ammonium nitrate in rice roots. (**A**) Venn diagram showing the number of differentially expressed genes for treatments with glutamine^35^, glutamate^36^, and ammonium nitrate (this study). (**B**) Quantitative RT-PCR analysis of *NAC5*, *MYB*, *LBD37*, *BBTI*, *KCS11*, *TAXI-I*, and an unknown gene. Seventeen-day-old nitrogen-starved rice seedlings were transferred to hydroponic solution containing 2.5 mM glutamine, glutamate, or 1.43 mM ammonium nitrate for 0–24 h^35,36^. Total RNA extracted from roots was used for qRT-PCR analysis. Relative expression indicates the fold-change of each gene as compared to that of control. Results are shown as means ± SD from three biological repeats.

We previously used microarray analysis to identify genes that were rapidly induced by 2.5 mM Gln or Glu^35,36^. Compared with the 158 genes identified in this study, ammonium nitrate induced 14 and 16 genes in common with Gln and Glu, respectively (Fig. 9A). These genes are listed in Supplementary Tables S4 and S5. The analysis revealed that only 7 genes were commonly induced by ammonium nitrate, Gln, and Glu (Fig. 9A). The proteins encoded by these 7 genes include three transcription factors, NAC5 (Os11g0184900), MYB (Os07g0119300), and LBD37 (Os07g0589000); two defense-related proteins, Bowman-Birk type trypsin inhibitor (BBTI, Os03g0823400) and xylanase inhibitor I-like protein (TAXI-I, Os05g0402900); one very long chain fatty acid biosynthetic enzyme, 3-ketoacyl-CoA synthase 11 (KCS11, Os02g0205500); and one protein of unknown function (Os02g0687200). We used qRT-PCR analysis to confirm that the expression of these 7 genes was rapidly (15 min–4 h) and strongly induced by ammonium nitrate, Gln and Glu (Fig. 9B).

Obertello *et al*. examined the effects of ammonium nitrate, at a much higher concentration (40 mM nitrate, 20 mM ammonium) and for a longer time (2 h), on the expression of rice genes^40^. In addition, a genome-wide transcriptome analysis of rice seedling roots in response to treatment with 1 mM ammonium for 3 h was recently reported by Chandran *et al*.^25^. We compared the 158 up-regulated genes identified here with the results from these two studies. Unexpectedly, the 158 genes induced by a low concentration ammonium nitrate (this study) shared only 27 and 38 genes with those reported by Obertello *et al*.^40^ and Chandran *et al*.^25^, respectively, and only 17 genes were shared by all three studies (Supplementary Fig. S6, Supplementary Tables S6–8). Interestingly, 8 of the 17 commonly induced genes encode proteins involved in nitrate/nitrite assimilation (NIA and NIR), ferredoxin reduction (Fd6, FdRB1, FNR, and FTR), and the pentose phosphate pathway (G6PDH and 6PGDH2) (Supplementary Table S8). *Os03g0764600*, encoding a MYB domain-containing protein, is the only transcription factor gene commonly induced by all three studies (Supplementary Table S8). These results suggest that our study identified a specific set of early N-responsive genes, especially those encoding transcription factors and protein kinases.

## Discussion

Ammonium has been considered the predominant nitrogen source for paddy field-grown rice^41^. However, the aerenchyma cells in rice roots may release oxygen to the rhizosphere, where ammonium can be converted to nitrate^32^. Several studies suggested that rice roots could efficiently take up nitrate formed by nitrification in the rhizosphere, and the rates of nitrate uptake were comparable to those of ammonium^33,42,43^. It is likely that rice roots grown under wetland conditions will simultaneously encounter both ammonium and nitrate. Therefore, we decided to study the effects of ammonium nitrate, rather than ammonium or nitrate alone, on rice seedlings. We are particularly interested in identifying genes that can rapidly respond to ammonium nitrate in rice roots. First, we used hydroponics to confirm that the optimal concentration for ammonium nitrate to support rice seedling growth was around 1–2.5 mM (Fig. 1) and adopted the concentration of 1.43 mM ammonium nitrate, as recommended by the International Rice Research Institute (IRRI), in this study^34^. The concentration of inorganic nitrogen in soil varies dramatically, ranging from a few hundred micromolar to around 20 mM or even higher, depending on soil type, microbial activity, and fertilizer addition *etc*.^44,45^. In contrast, the concentration of inorganic N inside the plant cell is less variable than that in soil. It has been estimated that the nitrate concentrations are 1–6 mM in the cytosol, and 5–75 mM in the vacuole^45^. Thus, the concentration of ammonium nitrate used in this study is of physiological significance.

The ammonium nitrate fed to N-starved rice seedlings will be assimilated into Gln and Glu via the GS/GOGAT cycle, where the amino acids serve as primary N donors for the synthesis of other N-containing compounds in the plant cell. Analysis of free amino acids in the roots of N-starved rice seedlings revealed that Gln was the only amino acid that rapidly accumulated after 15–30 min of ammonium nitrate treatment (Fig. 2A). The levels of Glu did not increase significantly until 8 h of ammonium nitrate treatment (Fig. 2B). It was somewhat unexpected that the amounts of Glu in the rice seedling roots decreased slightly within the first hour of ammonium nitrate treatment (Fig. 2B). Glu is a very active amino acid that serves as a precursor for many important metabolites in plants^36^. For instance, most of the transamination reactions require Glu as the N donor. The transfer of rice seedlings from N-deficient to N-sufficient conditions may quickly activate the synthesis of N-containing compounds, which includes many transamination reactions to consume Glu. It is possible that the demand for Glu was high during the early stage of ammonium nitrate treatment and the newly synthesized Glu was insufficient to meet the demand. Thus, steady-state levels of Glu decreased slightly within the first hour of ammonium nitrate treatment (Fig. 2B). Asp is another active amino acid that serves as a precursor for the synthesis of other amino acids and N-containing compounds. Similar to Glu, feeding of ammonium nitrate to N-starved rice seedlings resulted in a slight decrease in Asp levels within the first hour, but Asp started to increase after 8–16 h of treatment (Fig. 2C).

We also observed the rapid accumulation of Gln but not Glu in our previous studies when Gln or Glu was used to feed N-starved rice seedlings^35,36^. Unexpectedly, feeding of Glu to N-starved rice seedlings resulted in rapid accumulation of Gln but not Glu in the roots^36^. These results indicated that the exogenous Glu taken up by rice roots was quickly converted to the other N-containing compounds inside the plant cells^36^. Similarly, feeding of ammonium nitrate to N-starved rice seedlings for 15–30 min also resulted in rapid accumulation of Gln but not Glu. These results suggested that the absorbed ammonium nitrate was rapidly assimilated into Gln and Glu, and the newly synthesized Glu was also quickly converted into other N-containing compounds inside the plant cells. It is interesting that feeding of ammonium nitrate, Gln, or Glu to N-starved rice seedlings for 15–30 min resulted in significant increases in Gln but not Glu in the roots. The status of endogenous Gln has been proposed as a signal for N sufficiency in bacteria^46^. It is conceivable that plants may also use Gln as a signaling molecule to regulate metabolism, growth, and development^35^. Furthermore, these results also support the notion that the endogenous level of Glu is tightly regulated in plants^36^. It will be interesting to further investigate how the homeostasis of Glu is maintained in plants.

It is well known that ammonium and nitrate have synergistic effects in promoting rice growth^47^. However, the combinatorial effects of these two forms of inorganic N on the expression of rice genes has been less well studied. Here, we applied microarray and qRT-PCR analyses to identify genes that were rapidly regulated by ammonium nitrate (1.43 mM, 30 min) in rice roots. Interestingly, GO enrichment analysis revealed that the early ammonium nitrate-responsive genes identified in this study were related to “transcription factor activity”, “nucleus”, and “protein amino acid phosphorylation”.

We identified at least 35 early ammonium nitrate-responsive genes encoding transcription factors/nuclear proteins and several of these genes encode homologs of well-characterized N regulatory proteins. For instance, the Arabidopsis LBD37/38/39 proteins are involved in the regulation of N responses^37^. The expression of rice *LBD37/38/39* genes was rapidly and strongly induced by ammonium nitrate in the roots (Fig. 5 and Table 2). The maize Dof1 transcription factor is a positive regulator for N assimilation and plant growth^14,15^. The expression of rice *DOF4.6* (*Os04g0567800*) was also rapidly induced by ammonium nitrate (Supplementary Fig. S3 and Table 2). The MYB domain-containing proteins Os03g0764600 and Os07g0119300 belong to the G2-like transcription factor family subgroup HHO (for hypersensitivity to low phosphate-elicited primary root shortening1 [HRS1] homolog)^40^. The expression of these two genes was rapidly and strongly induced by ammonium nitrate (Fig. 5 and Supplementary Fig. S3). *Os03g0764600* has the highest number of connections in the Rice-Arabidopsis N-regulatory Network –Union analyzed by Obertello *et al*.^40^. Interestingly, *Os03g0764600* is also the only transcription factor gene commonly induced by ammonium^25^, and high^40^ and low (this study) concentrations of ammonium nitrate (Supplementary Table S8).

It is intriguing that several early ammonium nitrate-responsive transcription factor genes identified in this study are well known to be involved in stress signaling pathways. For instance, *NAC5* (*Os11g0184900*) is involved in abiotic stress tolerance^48–50^. The CIGR2 (Os07g0583600) transcription factor is involved in biotic stress responses^51^. The expression of *DREB1A* (*Os09g0522200*) was induced by drought and cold^52,53^. The expression of *RAP2-9* (*Os06g0166400*), which encodes an AP2 transcription factor, was also induced by drought^52^. The expression of these stress-related transcription factor genes was rapidly and strongly induced by ammonium nitrate (Fig. 5 and Supplementary Fig. S3). The involvement of these genes in the regulation of N responses has yet to be characterized in rice. Most of the transcription factor genes identified here are of unknown function (Table 2). It is likely that we have identified novel regulatory proteins involved in N responses. Some of the transcription factors identified here may play important roles in N sensing and signaling in rice.

In addition to transcription factors/nuclear proteins, we have identified several early ammonium nitrate-responsive genes encoding MAPKKK and CIPK proteins (Fig. 6 and Table 2). The MAP kinase cascade is involved in biotic and abiotic stress responses in plants^54^. However, the involvement of MAPK in N sensing and signaling is less studied. In Arabidopsis, MEKK1 is involved in Glu signaling^55^ and MKK9 modulates N acquisition under N-limiting conditions^56^. Arabidopsis CIPK8 is involved in early nitrate signaling^38^, whereas CIPK23 regulates the activity of ammonium and nitrate transporters^39,57^. We used qRT-PCR to confirm that the expression of 6 rice *MAPKKK* genes, e.g. *MAPKKK55, 62–64, 69*, and 70, was rapidly and strongly induced by ammonium nitrate in the roots (Fig. 6). The functions of these *MAPKKK* genes have yet to be characterized. As the functions of most protein kinases/phosphatases identified here are still unknown, it will be interesting to further investigate if these protein kinases/phosphatases are directly involved in the regulation of N signaling pathways in rice.

It is interesting that feeding of ammonium nitrate to N-starved rice seedlings also rapidly induced the expression of genes involved in defense/stress responses. In addition to the abovementioned *DREB1A*, *NAC5*, *RAP2-9*, *CIGR2*, and *MAPKKKs*, ammonium nitrate also rapidly induced the expression of many stress-responsive genes, such as *BBTI*, *KCS11*, and *TAXI-I* (Fig. 8). This is reminiscent of our previous studies on the identification of early Gln- and Glu-responsive genes in rice roots^35,36^. We have shown that feeding of Gln or Glu to N-starved rice seedlings can rapidly induce the expression of metabolic and stress/defense-responsive genes^35,36^. Here, we found that the expression of many genes involved in metabolism and stress/defense responses was also rapidly induced by ammonium nitrate. It seems that feeding of ammonium nitrate, Gln, or Glu to N-starved rice seedlings shares a common theme in the activation of metabolic and stress/defense-related genes. Thus, N nutrients not only promote plant growth and development but also rapidly equip plants with stress/defense genes to cope with adverse environments. These findings suggest that N nutrition and its signaling pathways are interconnected with stress/defense responses, regardless of the form of N. The identification of early ammonium nitrate-, Gln-, and Glu-responsive transcription factor and protein kinase/phosphatase genes provides useful information for future studies on the crosstalk between N and defense/stress signaling pathways in plants.

Transcriptomic analyses revealed that feeding of ammonium nitrate (1.43 mM), Glu (2.5 mM) and Gln (2.5 mM) to N-starved rice seedlings for 30 min rapidly induced the expression of 158, 124, and 35 genes in the roots, respectively (Fig. 8A). These results indicate that ammonium nitrate and Glu have more profound effects than Gln on the induction of gene expression in rice roots. In addition to their roles as metabolic fuels, the signaling functions of nitrate and Glu are widely accepted in plants. By contrast, the signaling role of Gln is less well-established in plants. Although the total number of Gln-induced genes is small, 10 of the 35 early Gln-responsive genes encode transcription factors^35^. Interestingly, some of the early Gln-responsive transcription factor genes play important roles in the regulation of N and stress responses^35^. These results suggest that Gln may function as a metabolic fuel and a signaling molecule in plants. Feeding of ammonium nitrate to N-starved rice seedlings resulted in rapid accumulation of Gln in the roots within 15 min (Fig. 2) indicating that the absorbed ammonium nitrate was quickly converted to Gln and Glu via the GS/GOGAT cycle. This raises the interesting question of whether some of the early ammonium nitrate-responsive genes may be actually responsive to Gln and/or Glu. Comparisons among our transcriptomic data derived from rice roots revealed that early ammonium nitrate-responsive genes shared only 14 and 16 genes with those of Gln and Glu, respectively. The majority of early ammonium nitrate-responsive genes, e.g. 135 out of 158, are specifically induced by ammonium nitrate (Fig. 8A). Similarly, most of the early Glu-responsive genes (96 out of 124) are specific to Glu (Fig. 8A). These results suggest that ammonium nitrate, Glu, and Gln are highly specific for the induction of their early responsive genes. Therefore, the indirect effects of ammonium nitrate mediated by Gln and/or Glu on the induction of early responsive genes were limited in rice roots.

Still, we identified 7 genes, *NAC5* (*Os11g0184900*), *MYB* (*Os07g0119300*), *LBD37* (*Os07g0589000*), *BBTI* (*Os03g0823400*), *TAXI-I* (*Os05g0402900*), *KCS11* (*Os02g0205500*), and an unknown gene (*Os02g0687200*), that are commonly induced by ammonium nitrate, Gln, and Glu in rice roots (Fig. 8A). The expression of *NAC5* (*Os11g0184900*) and *MYB* (*Os07g0119300*) was preferentially induced by ammonium nitrate and the expression of *LBD37* (*Os07g0589000*) was preferentially induced by Gln (Fig. 8B). Nevertheless, the expression of these 7 genes was rapidly and strongly induced by ammonium nitrate, Gln, and Glu (Fig. 8B). It is tempting to hypothesize that these genes may be involved in the regulation of more general N signals. Interestingly, the functions of these genes are either related to N signaling (*LBD37* and *MYB*) or stress/defense responses (*BBTI*, *TAXI-I*, and *KCS11*), which coincide with the common theme of early ammonium nitrate-, Gln-, and Glu-responsive genes discussed above.

Plants encounter different forms of N in nature and therefore, mechanisms to perceive specific form of N and generate specific responses can help plants adapt to the changing environment. The nutritional effects of ammonium nitrate on rice have been recognized for a long time. We recently demonstrated that the first organic N compounds derived from the primary N assimilation pathway, e.g. Gln and Glu, were also very effective at supporting rice seedling growth^35,36^. In support of their respective nutritional effects, feeding of ammonium nitrate, Gln, and Glu to N-starved rice seedlings rapidly induced the expression of genes specifically involved in the transport and metabolism of different forms of N. Moreover, ammonium nitrate, Gln, and Glu also exert their signaling functions to activate the expression of specific sets of genes. We have identified several N-responsive regulatory genes and stress-responsive genes that are specifically induced by ammonium nitrate in this study. In addition, we have uncovered a few candidate genes that are commonly induced by ammonium nitrate, Gln, and Glu. The discovery of these specific and general N regulatory genes provides useful information for further dissecting the molecular mechanisms of N signaling pathways and their interactions with stress/defense responses in rice.

## Methods

### Plant material and growth conditions

Rice (*Oryza sativa* L. ssp. *japonica* cv. TNG67) seeds were germinated in darkness at 30 °C for 3 days. After germination, the etiolated rice seedlings were transferred to hydroponic solutions^34^ without (−N) or supplemented with 0.1–10 mM NH_4_NO_3_ in a controlled growth chamber at 30 °C under a 12-h light/12-h dark photoperiod with 200 µmol photons m^−2^ s^−1^ light intensity and 70% relative humidity for 2 weeks. The hydroponic solution was renewed every 3 days in all experiments.

### Measurement of chlorophyll content

The Chlorophyll Content Meter (CCM-300, Opti-sciences, NH, USA) was used to measure the amount of chlorophyll in the leaves of 17-day-old rice seedlings grown in hydroponic solutions −N or supplemented with 0.1–10 mM NH_4_NO_3_. Fifteen biological replicates with 3 technical repeats from each treatment were used for chlorophyll measurement.

### RNA isolation and microarray analysis

Total RNA extracted from roots of 17-day-old rice seedlings grown in hydroponic solution −N or +1.43 mM NH_4_NO_3_ for 30 min (+N) was used for microarray analysis with the GeneChip Rice Genome Array (Affymetrix, Santa Clara, CA, USA). The method for total RNA isolation was as described previously^58^. RNA samples from two biological repeats were used for the microarray experiment conducted by the Affymetrix Gene Expression Service Lab at Academia Sinica, Taipei, Taiwan (http://ipmb.sinica.edu.tw/affy/). Target preparation, hybridization, washes, staining, array scanning, and data analysis were performed as described^35^. Two-fold cutoff and a *p*-value less than 0.05 were applied to select for up- and down-regulated genes after 1.43 mM NH_4_NO_3_ treatment for 30 min. AgriGO (http://bioinfo.cau.edu.cn/agriGO/) was used to perform the gene ontology (GO) analysis of 158 NH_4_NO_3_ up-regulated genes compared with the genome-wide background with an adjusted *p*-value (False Discovery Rate, FDR) cutoff of 0.05. The GO categories consisting of three structured networks, e.g. biological process, cellular component and molecular function, of defined terms were derived from Gene Ontology (http://www.geneontology.org). The nomenclature of rice MAPKKKs listed in this study was according to Rao *et al*.^59^. A web-based program EXPath (http://expath.itps.ncku.edu.tw) was used to analyze KEGG pathway enrichment with the thresholds of *p*-value < 0.05.

### Quantitative RT-PCR analysis of NH_4_NO_3_-responsive genes

To examine the effect of NH_4_NO_3_ on the expression of NH_4_NO_3_-responsive genes, 17-day-old rice seedlings grown in -N hydroponic solution were transferred to solutions containing 1.43 mM NH_4_NO_3_ for 0–24 h. Total RNA extracted from roots and shoots of NH_4_NO_3_-treated rice seedlings was digested with DNase I and used for qRT-PCR analysis. All of the expression data from root samples were normalized to the nuclear gene *UBC3* (*Os02g0634800*). Two reference genes, *UBC3* (*Os02g0634800*) and *UBQ10* (*Os02g 0161900*), were used to normalize the expression data for shoot samples. The primers used for qRT-PCR analysis are listed in Supplementary Table S9. The cDNA sequences of *CIPK14* and *CIPK15* are very similar and the primers used in qRT-PCR analysis cannot distinguish these two genes. The qRT-PCRs were performed in triplicate for each sample in three independent experiments. The expression of genes listed in Supplementary Table S1 was up- or down-regulated by ammonium nitrate (1.43 mM, 30 min) for more than 2-fold in the qRT-PCR analysis.

### Amino acid analysis

Seventeen-day-old rice seedlings grown in -N hydroponic solution were transferred to fresh −N or −N supplemented with 1.43 mM NH_4_NO_3_ for 0–24 h. The method for amino acid extraction from rice roots was described previously^35^. Amino acid samples from four biological repeats were analyzed using the Waters Acquity UPLC system equipped with a Waters AccQ•Tag Ultra column (2.1 mm × 10 mm, 1.7 μm particles) as previously described^35^.

### Data Availability

All data generated or analyzed during this study are included in this published article and its Supplementary Information files. The microarray datasets generated and analyzed during the current study are available in the NCBI GEO repository GSE98017 (https://www.ncbi.nlm.nih.gov/geo/query/acc.cgi?acc = GSE98017).

## Electronic supplementary material

Supplementary Information
