# Supplementary material for: Identification of early ammonium nitrate-responsive genes in rice roots
Source: Sci Rep. 2017 Dec 4;7:16885. doi: 10.1038/s41598-017-17173-9 (PMC5715151; doi:10.1038/s41598-017-17173-9)
Supplement: Supplementary file 1 — Supplementary Information [file 41598_2017_17173_MOESM1_ESM.pdf]

Identification of early ammonium nitrate-responsive genes in rice roots

Hsiu-Chun Yang[†], Chia-Cheng Kan[†], Tze-Huan Hung[†], Ping-Han Hsieh[†], Shi-Yun Wang, Wei-Yu Hsieh and Ming-Hsiun Hsieh*

Supplementary Information

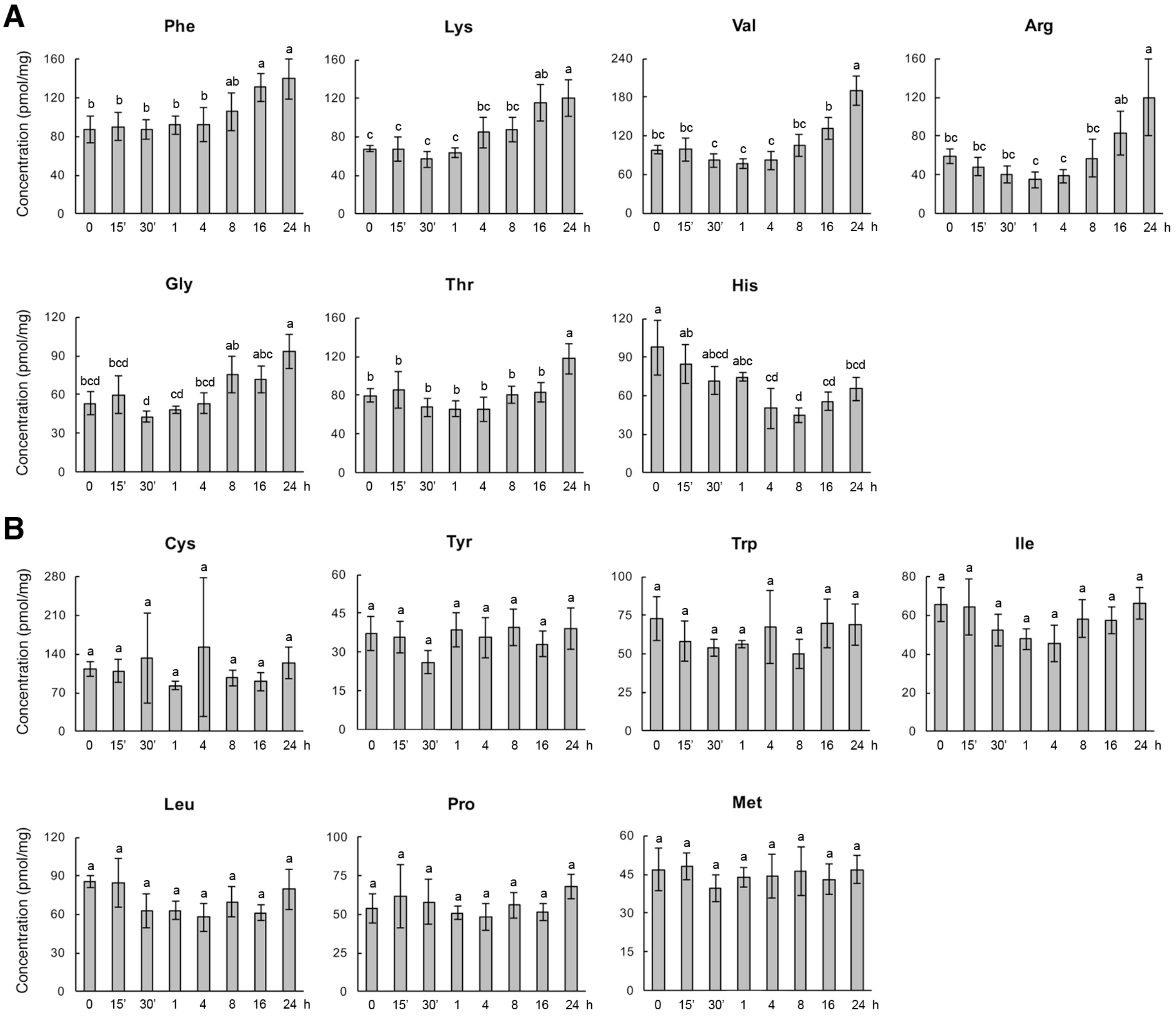

Fig. S1 Amino acid contents in the rice roots during time course of ammonium nitrate treatment. (**A**) Contents of phenylalanine, lysine, valine, arginine, glycine, and threonine increased slightly in the roots of 17-day-old nitrogen-starved rice seedlings after 8–24 h of 1.43 mM ammonium nitrate treatment. The contents of histidine decreased slightly after 4–24 h of ammonium nitrate treatment. **(B)** Feeding of ammonium nitrate did not affect the contents of cysteine, tyrosine, tryptophan, isoleucine, leucine, proline and methionine in rice roots. Data are means ± SD (n = 4). Different letters indicate significant differences between treatments, tested by one-way ANOVA followed by Tukey's test (P < 0.05).

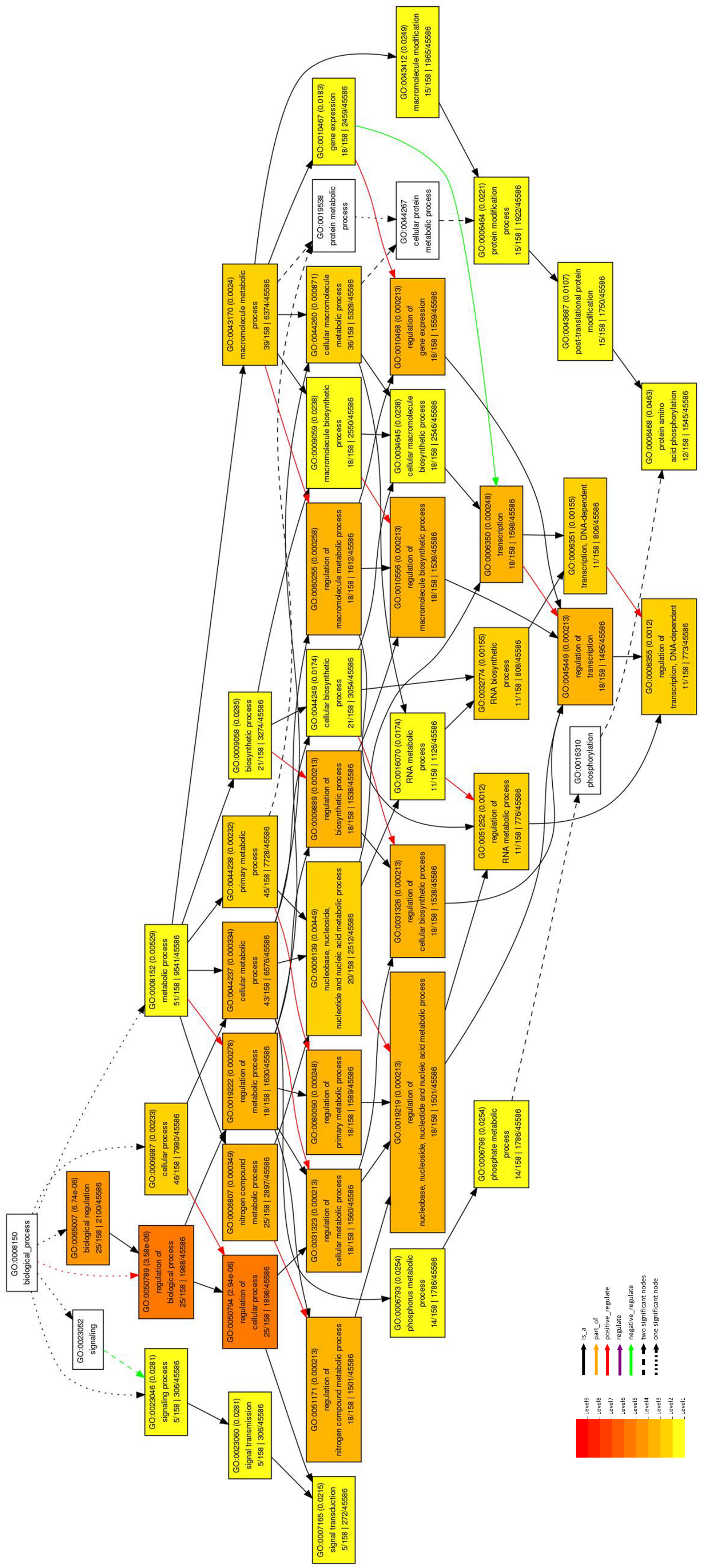

Fig. S2 Gene ontology enrichment analysis of genes up-regulated by ammonium nitrate. The differentially expressed genes were analyzed by singular enrichment analysis using AgriGO. Significantly enriched GO categories in biological function are shown in yellow, orange and red (false discovery rate, FDR < 0.05).

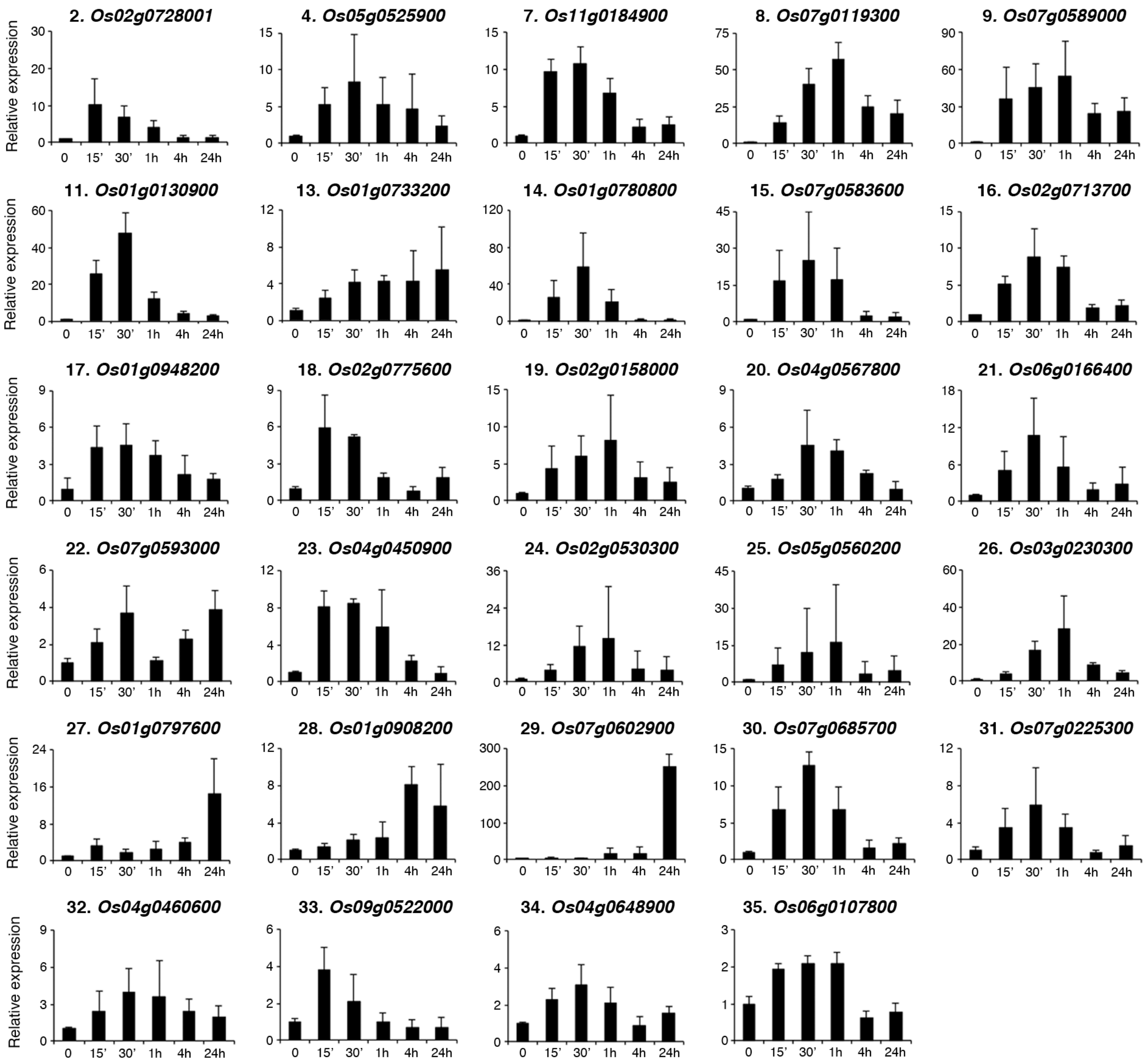

Fig. S3 Quantitative RT-PCR analysis of genes encoding putative transcription factors/nuclear proteins that are rapidly induced by ammonium nitrate identified in this study. Total RNA extracted from roots of 17-day-old rice seedlings treated with ammonium nitrate for 0–24 h was used for qRT-PCR analysis. Relative expression indicates the fold-change of each gene as compared to that of control. The number of each gene corresponds to the number in Table 2. Results are shown as means ± SD from three biological repeats.

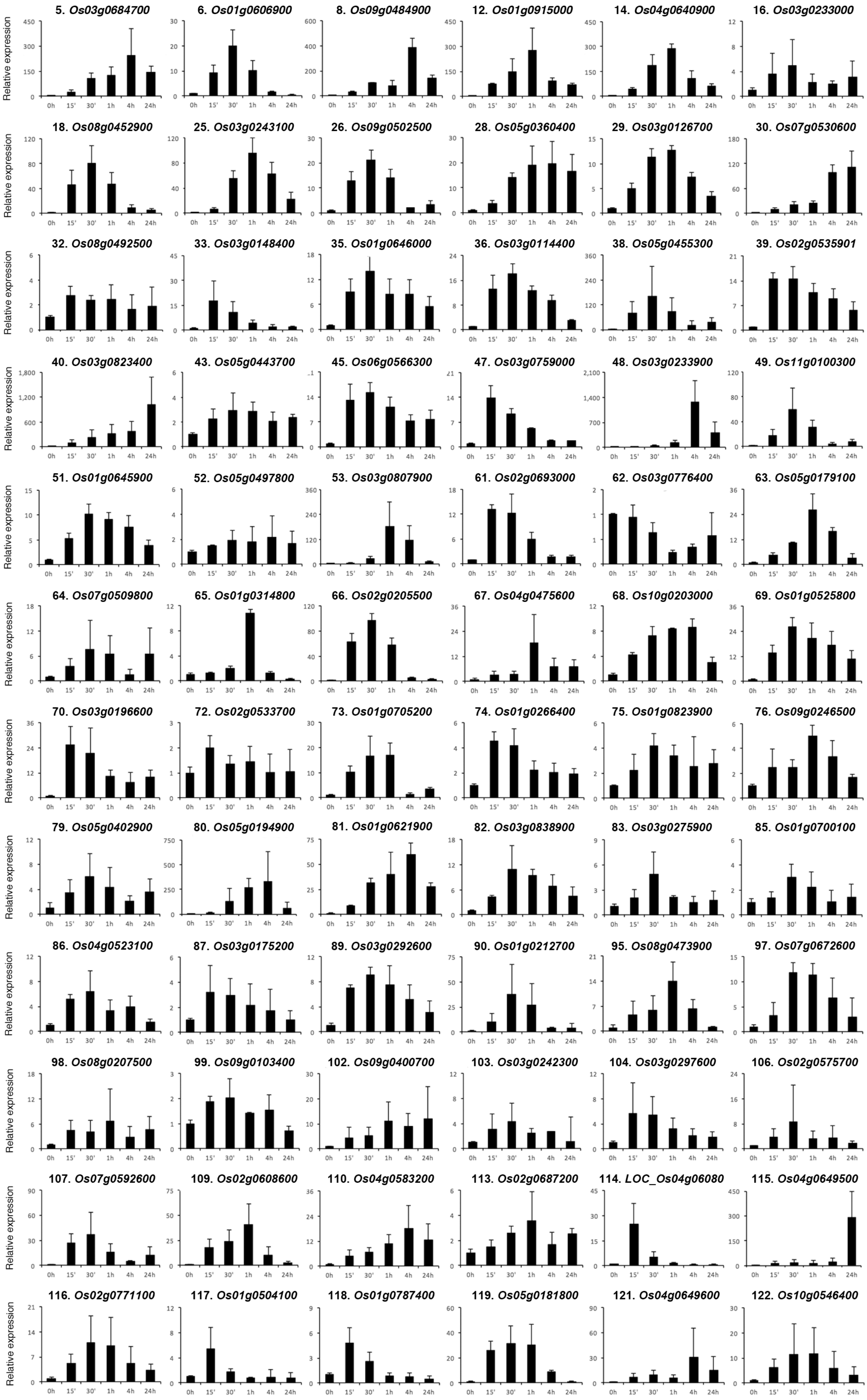
5. Os03g0684700
6. Os01g0606900
8. Os09g0484900
12. Os01g0915000
14. Os04g0640900
16. Os03g0233000
18. Os08g0452900
25. Os03g0243100
26. Os09g0502500
28. Os05g0360400
29. Os03g0126700
30. Os07g0530600
32. Os08g0492500
33. Os03g0148400
35. Os01g0646000
36. Os03g0114400
38. Os05g0455300
39. Os02g0535901
40. Os03g0823400
43. Os05g0443700
45. Os06g0566300
47. Os03g0759000
48. Os03g0233900
49. Os11g0100300
51. Os01g0645900
52. Os05g0497800
53. Os03g0807900
61. Os02g0693000
62. Os03g0776400
63. Os05g0179100
64. Os07g0509800
65. Os01g0314800
66. Os02g0205500
67. Os04g0475600
68. Os10g0203000
69. Os01g0525800
70. Os03g0196600
72. Os02g0533700
73. Os01g0705200
74. Os01g0266400
75. Os01g0823900
76. Os09g0246500
79. Os05g0402900
80. Os05g0194900
81. Os01g0621900
82. Os03g0838900
83. Os03g0275900
85. Os01g0700100
86. Os04g0523100
87. Os03g0175200
89. Os03g0292600
90. Os01g0212700
95. Os08g0473900
97. Os07g0672600
98. Os08g0207500
99. Os09g0103400
102. Os09g0400700
103. Os03g0242300
104. Os03g0297600
106. Os02g0575700
107. Os07g0592600
109. Os02g0608600
110. Os04g0583200
113. Os02g0687200
114. LOC_Os04g06080
115. Os04g0649500
116. Os02g0771100
117. Os01g0504100
118. Os01g0787400
119. Os05g0181800
121. Os04g0649600
122. Os10g0546400
Relative expression
0h
15'
30'
1h
4h
24h

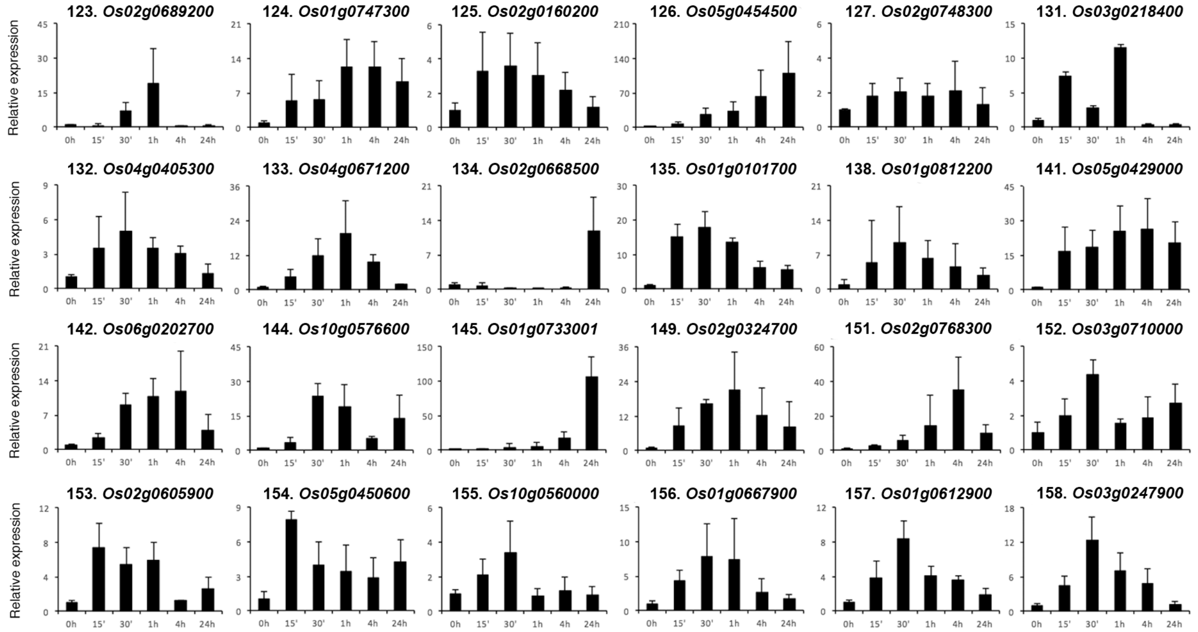

Fig. S4 Quantitative RT-PCR analysis of early ammonium nitrate-responsive genes identified in this study. Total RNA extracted from roots of 17-day-old rice seedlings treated with 1.43 mM ammonium nitrate for 0–24 h was used for qRT-PCR analysis. Relative expression indicates the fold-change of each gene as compared to that of control. The number of each gene corresponds to the number in Table S1. Results are shown as means ± SD from three biological repeats.

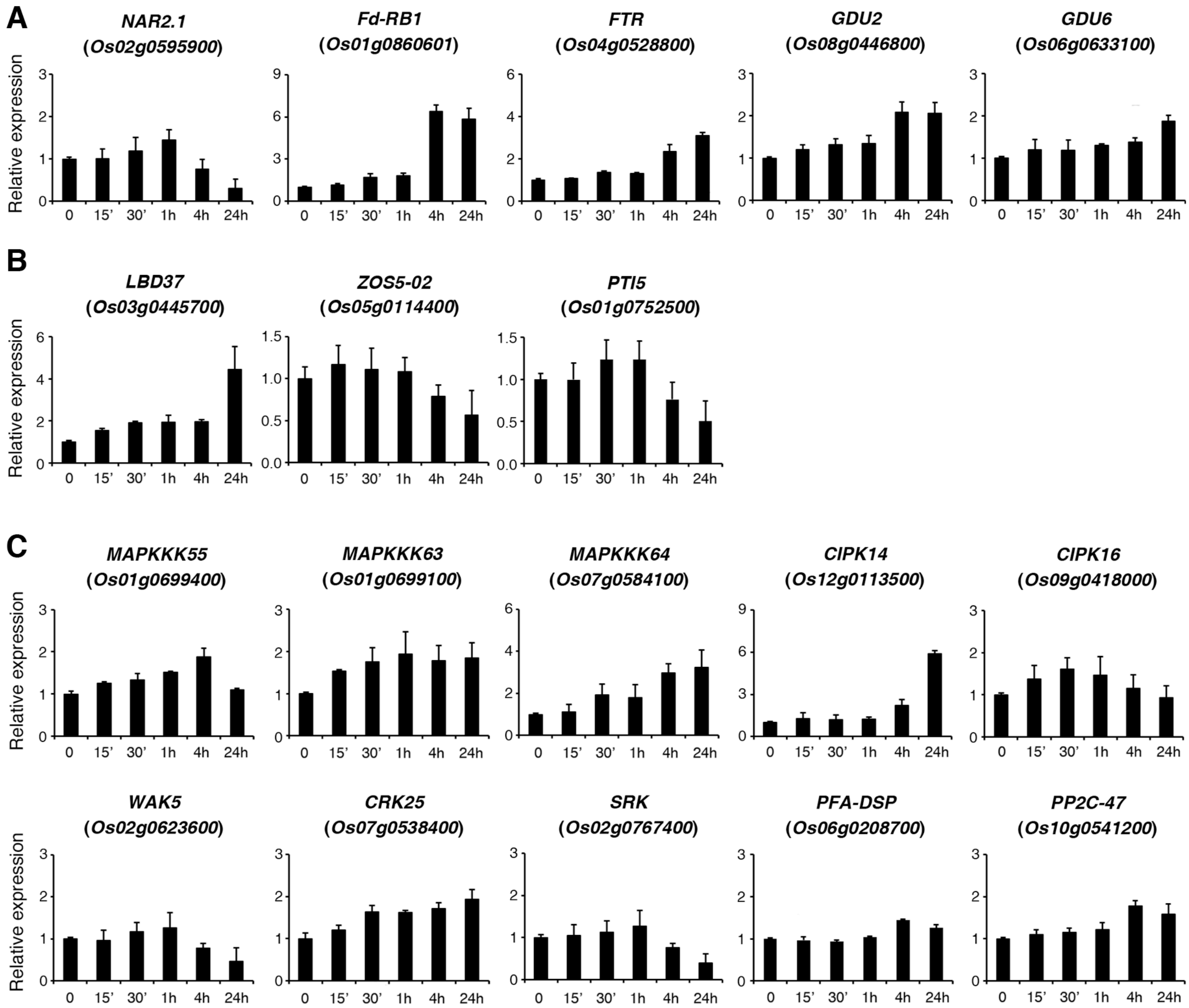

Fig. S5 Quantitative RT-PCR analysis of early ammonium nitrate-responsive genes in rice shoots. Total RNA extracted from shoots of 17-day-old rice seedlings treated with 1.43 mM ammonium nitrate for 0–24 h was used for qRT-PCR analysis. Relative expression indicates the fold-change of each gene as compared to that of control. Two reference genes, *UBC3* (*Os02g0634800*) and *UBQ10* (*Os02g 0161900*), were used to normalize the expression data. Results are shown as means ± SD from three biological repeats.

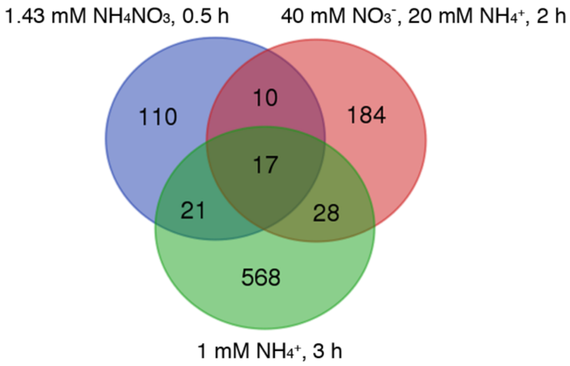

Fig. S6 Venn diagram of genes commonly induced by ammonium and ammonium nitrate. The numbers are up-regulated genes in rice roots identified by Chandran *et al.*[25], Obertello *et al.*[40], and this study. Obertello *et al.*[40] treated rice seedlings with 40 mM nitrate and 20 mM ammonium for 2 hours to identify differentially expressed genes. Chandran *et al.*[25] used 1 mM ammonium to treat rice seedlings for 3 hours to identify nitrogen-responsive genes.

Table S1. List of early ammonium nitrate-responsive genes in rice roots

No.	Locus ID		Fold change (+ NH_4NO_3/-N)	Gene description
1	Os02g0770800	LOC_Os02g53130	65.8	Nitrate reductase [NAD(P)H]
2	Os01g0860601	LOC_Os01g64120	42.2	Ferredoxin, root R-B1
3	Os01g0631200	LOC_Os01g44050	32.3	Uroporphyrinogen-III C-methyltransferase
4	Os03g0609500	LOC_Os03g41330	30.5	LOB domain-containing protein 38 (LBD38)
5	Os03g0684700	LOC_Os03g48030	25.8	Unknown, HPP family protein
6	Os01g0606900	LOC_Os01g42190	20.5	DnaJ homolog subfamily B member 7
7	Os02g0728001	LOC_Os02g49560	17.2	Basic leucine zipper 43-like (bZIP 43-like)
8	Os09g0484900	LOC_Os09g31130	15.7	Dicarboxylate transporter, solute carrier 13 (SLC13)
9	Os03g0445700	LOC_Os03g33090	15.0	LOB domain-containing protein 37 (LBD37)
10	Os05g0525900	LOC_Os05g45020	14.7	Zinc finger CCCH domain-containing protein 37
11	Os09g0522200	LOC_Os09g35030	14.7	Dehydration-responsive element-binding protein 1A (DREB1A)
12	Os01g0915000	LOC_Os01g68650	14.4	Unknown, DUF506
13	Os05g0114400	LOC_Os05g02390	14.0	Zinc finger protein, ZOS5-02
14	Os04g0640900	LOC_Os04g54830	13.4	Unknown
15	Os11g0184900	LOC_Os11g08210	11.5	NAC domain-containing protein, NAC5
16	Os03g0233000	LOC_Os03g13050	10.4	Calcium uniporter protein 4, mitochondrial
17	Os07g0119300	LOC_Os07g02800	9.4	MYB family protein
18	Os08g0452900	LOC_Os08g35160	9.4	DnaJ/heat shock protein 40
19	Os07g0589000	LOC_Os07g40000	8.5	LOB domain-containing protein 37 (LBD37)
20	Os03g0784700	LOC_Os03g57120	8.5	Ferredoxin--NADP reductase (FNR)
21	Os01g0699100	LOC_Os01g50370	8.2	Mitogen-activated protein kinase kinase kinase 63 (MAPKKK63)
22	Os03g0764600	LOC_Os03g55590	8.0	MYB family protein
23	Os01g0357100	LOC_Os01g25484	8.0	Nitrite reductase
24	Os04g0480200	LOC_Os04g40410	7.0	High-affinity nitrate transporter 2.2 (NAR2.2)
25	Os03g0243100	LOC_Os03g13950	6.5	Actin-depolymerizing factor 5 (ADF5)
26	Os09g0502500	LOC_Os09g32570	6.5	Quinone-oxidoreductase homolog
27	Os01g0130900	LOC_Os01g03980	6.2	Zinc finger protein ZIC 2
28	Os05g0360400	LOC_Os05g29710	5.9	E3 ubiquitin-protein ligase EL5-like
29	Os03g0126700	LOC_Os03g03500	5.7	Unknown, heavy metal associated protein (HMA)
30	Os07g0530600	LOC_Os07g34640	5.4	Pyruvate, phosphate dikinase regulatory protein (PDRP1)
31	Os11g0113700	LOC_Os11g02240	5.4	CBL-interacting protein kinase 15 (CIPK15)
	Os12g0113500	LOC_Os12g02200		CBL-interacting protein kinase 14 (CIPK14)
32	Os08g0492500	LOC_Os08g38460	5.3	Probable E3 ubiquitin-protein ligase XERICO
33	Os03g0148400	LOC_Os03g05460	5.3	Unknown, DUF740
34	Os02g0595900	LOC_Os02g38230	5.2	High-affinity nitrate transporter 2.1 (NAR2.1)
35	Os01g0646000	LOC_Os01g45840	5.1	Pentatricopeptide repeat (PPR) protein
36	Os03g0114400	LOC_Os03g02330	5.1	AAA-ATPase
37	Os07g0406300	LOC_Os07g22350	5.1	Glucose-6-phosphate 1-dehydrogenase 2

38	Os05g0455300	LOC_Os05g38130	5.0	Unknown
39	Os02g0535901	LOC_Os02g33240	5.0	Probable F-box protein
40	Os03g0823400	LOC_Os03g60840	5.0	Bowman-Birk type trypsin inhibitor
41	Os01g0752500	LOC_Os01g54890	5.0	Pathogenesis-related genes transcriptional activator (PTI5)
42	Os06g0633100	LOC_Os06g42660	4.9	Glutamine dumper 6 (GDU6)
43	Os05g0443700	LOC_Os05g37150	4.8	Unknown, Syntaxin 6 N-terminal domain-containing protein
44	Os01g0733200	LOC_Os01g53220	4.7	Heat stress transcription factor C-1b
45	Os06g0566300	LOC_Os06g37010	4.5	Zinc transporter 10 (ZIP10)
46	Os11g0484500	LOC_Os11g29400	4.3	6-Phosphogluconate dehydrogenase 2 (6PGDH2)
47	Os03g0759000	LOC_Os03g55180	4.2	Unknown, DUF1336
48	Os03g0233900	LOC_Os03g13140	4.2	Non-symbiotic hemoglobin 1 (HB1)
49	Os11g0100300	LOC_Os11g01030	4.1	Unknown
50	Os01g0780800	LOC_Os01g57240	4.1	ULTRAPETALA 1 (ULT1), trithorax group factor
51	Os01g0645900	LOC_Os01g45830	4.1	Molybdate transporter 2 (MOT2); sulfate transporter (SUL1)
52	Os05g0497800	None	4.0	Unknown
53	Os03g0807900	LOC_Os03g59320	3.9	Unknown
54	Os07g0583600	LOC_Os07g39470	3.8	Chitin-inducible gibberellin-responsive protein 2 (CIGR2), GRAS family protein
55	Os02g0713700	LOC_Os02g48320	3.6	AT-hook motif nuclear-localized protein 25
56	Os01g0948200	LOC_Os01g71970	3.6	Scarecrow-like protein 3; GRAS family protein
57	Os01g0699600	LOC_Os01g50420	3.6	Mitogen-activated protein kinase kinase kinase 62 (MAPKKK62)
58	Os02g0623600	LOC_Os02g41480	3.5	Wall-associated receptor kinase 5
59	Os02g0775600	LOC_Os02g53530	3.5	Zinc finger protein 8
60	Os09g0418000	LOC_Os09g25090	3.5	CBL-interacting protein kinase 16 (CIPK16)
61	Os02g0693000	LOC_Os02g46620	3.5	Unknown
62	Os03g0776400	LOC_Os03g56500	3.5	Unknown
63	Os05g0179100	LOC_Os05g08620	3.3	Unknown
64	Os07g0509800	LOC_Os07g32570	3.3	Adenosine 5'-phosphosulfate reductase 1 (APR1)
65	Os01g0314800	LOC_Os01g21250	3.2	Late embryogenesis abundant protein Lea5-D
66	Os02g0205500	LOC_Os02g11070	3.1	3-Ketoacyl-CoA synthase 11
67	Os04g0475600	LOC_Os04g39980	3.1	2-Oxoglutarate-dependent-Fe (II) dioxygenase; dioxygenase for auxin oxidation (DAO)
68	Os10g0203000	LOC_Os10g13550	3.1	EF-hand domain calcium-binding protein
69	Os01g0525800	None	3.1	NADH-ubiquinone oxidoreductase chain 1-like
70	Os03g0196600	LOC_Os03g10050	3.1	Serine acetyltransferase 4 (SAT4)
71	Os02g0158000	LOC_Os02g06330	3.0	AP2/ERF transcription factor
72	Os02g0533700	LOC_Os02g33060	3.0	Unknown
73	Os01g0705200	LOC_Os01g50910	3.0	Late embryogenesis abundant protein, group 3
74	Os01g0266400	LOC_Os01g16140	2.9	Unknown
75	Os01g0823900	LOC_Os01g60860	2.9	U-box domain-containing protein 16
76	Os09g0246500	LOC_Os09g07150	2.9	Unknown

77	Os01g0699400	LOC_Os01g50400	2.9	Mitogen-activated protein kinase kinase kinase 55 (MAPKKK55)
78	Os07g0584100	LOC_Os07g39520	2.9	Mitogen-activated protein kinase kinase kinase 64 (MAPKKK64)
79	Os05g0402900	LOC_Os05g33400	2.9	Xylanase inhibitor I-like (TAXI-I), basic 7S globulin
80	Os05g0194900	LOC_Os05g10650	2.9	ATP-dependent 6-phosphofructokinase 6
81	Os01g0621900	LOC_Os01g43370	2.9	uORF-containing transcript
82	Os03g0838900	LOC_Os03g62240	2.9	Unknown, mTERF domain-containing protein
83	Os03g0275900	LOC_Os03g16824	2.9	Spotted leaf 11 (SPL11), U-box-ARM protein
84	Os05g0443500	LOC_Os05g37140	2.9	Ferredoxin-6
85	Os01g0700100	LOC_Os01g50460	2.9	Sugar transporter SWEET2b
86	Os04g0523100	LOC_Os04g44210	2.9	Unknown, DUF3755
87	Os03g0175200	LOC_Os03g07890	2.8	Solute carrier family 25 member 44, mitochondrial carrier protein
88	Os04g0567800	LOC_Os04g47990	2.8	Dof zinc finger protein DOF4.6
89	Os03g0292600	None	2.8	Unknown
90	Os01g0212700	LOC_Os01g11460	2.8	RING-H2 finger protein ATL39
91	Os06g0166400	LOC_Os06g07030	2.8	Ethylene-responsive transcription factor RAP2-9
92	Os05g0545400	LOC_Os05g46760	2.7	Mitogen-activated protein kinase kinase kinase 69 (MAPKKK69)
93	Os07g0593000	LOC_Os07g40300	2.7	Zinc finger protein 7 (ZFP7)
94	Os04g0450900	LOC_Os04g37790	2.7	Myosin heavy chain, SMC chromosome segregation protein
95	Os08g0473900	LOC_Os08g36910	2.7	Alpha-amylase isozyme 3D (AMY3D)
96	Os01g0292200	LOC_Os01g18800	2.7	CBL-interacting protein kinase 1 (CIPK1)
97	Os07g0672600	LOC_Os07g47540	2.7	LURP-one-related (LOR) 2-like, Tubby C2
98	Os08g0207500	LOC_Os08g10630	2.6	Zinc transporter 4 (ZIP4)
99	Os09g0103400	LOC_Os09g01610	2.6	WASH complex subunit CCDC53 homolog
100	Os02g0530300	LOC_Os02g32840	2.6	Zinc finger A20, AN1 domain-containing stress-associated protein 5 (SAP5)
101	Os05g0560200	LOC_Os05g48650	2.5	Seed dormancy control (DOG1), transcription factor-like protein
102	Os09g0400700	LOC_Os09g23590	2.5	Unknown
103	Os03g0242300	LOC_Os03g13870	2.5	Formin-like protein 5
104	Os03g0297600	LOC_Os03g18600	2.5	Abscisic acid receptor PYL4
105	Os06g0208700	LOC_Os06g10650	2.5	Plant and fungi atypical dual-specificity phosphatase (PFA-DSP)
106	Os02g0575700	LOC_Os02g36590	2.5	Loricrin
107	Os07g0592600	LOC_Os07g40290	2.5	Indole-3-acetic acid-amido synthetase GH3.8
108	Os03g0230300	LOC_Os03g12820	2.5	RST and PARP domain-containing SRO (similar to RCD one) protein (SRO2)
109	Os02g0608600	LOC_Os02g39570	2.4	Acetolactate synthase small subunit 2
110	Os04g0583200	LOC_Os04g49370	2.4	Unknown
111	Os01g0797600	LOC_Os01g58420	2.4	Ethylene-responsive transcription factor 8
112	Os01g0908200	LOC_Os01g68020	2.4	BTB/POZ and TAZ domain-containing protein 2
113	Os02g0687200	LOC_Os02g46210	2.3	Unknown, DUF581
114	Os.22947.1.S1_at	LOC_Os04g06080	2.3	Retrotransposon protein
115	Os04g0649500	LOC_Os04g55600	2.3	Unknown

116	Os02g0771100	LOC_Os02g53140	2.3	E3 ubiquitin-protein ligase COP1
117	Os01g0504100	LOC_Os01g31940	2.3	Purine permease 4
118	Os01g0787400	LOC_Os01g57740	2.3	Unknown
119	Os05g0181800	None	2.3	Unknown
120	Os07g0602900	LOC_Os07g41160	2.3	Ninja-family protein
121	Os04g0649600	LOC_Os04g55610	2.3	Unknown
122	Os10g0546400	LOC_Os10g39920	2.3	Cyclin-dependent protein kinase inhibitor SMR1
123	Os02g0689200	LOC_Os02g46380	2.3	Purine permease 11
124	Os01g0747300	LOC_Os01g54340	2.2	Unknown, DUF506
125	Os02g0160200	LOC_Os02g06520	2.2	F-box protein
126	Os05g0454500	LOC_Os05g38040	2.2	Unknown
127	Os02g0748300	LOC_Os02g51350	2.2	F-box/kelch-repeat protein
128	Os01g0699500	LOC_Os01g50410	2.2	Mitogen-activated protein kinase kinase kinase 70 (MAPKKK70)
129	Os07g0538400	LOC_Os07g35390	2.2	Cysteine-rich receptor-like protein kinase 25
130	Os10g0541200	LOC_Os10g39540	2.2	Phosphatase 2C 47
131	Os03g0218400	LOC_Os03g11900	2.2	Sugar transport protein 13
132	Os04g0405300	LOC_Os04g33240	2.2	Phytoalexin momilactone A synthase-like
133	Os04g0671200	LOC_Os04g57550	2.2	Polyamine oxidase 2
134	Os02g0668500	LOC_Os02g44820	2.1	Ganglioside-induced differentiation-associated protein 2, Sec14p-like lipid-binding domain-containing protein
135	Os01g0101700	LOC_Os01g01160	2.1	Chaperone protein dnaJ 20, dnaJ/Hsp40
136	Os07g0685700	LOC_Os07g48630	2.1	ETHYLENE INSENSITIVE 3-like 1 protein, EIL2
137	Os07g0225300	LOC_Os07g12340	2.1	NAC domain-containing protein 67
138	Os01g0812200	LOC_Os01g59690	2.1	F-box protein
139	Os04g0528800	LOC_Os04g44650	2.1	Ferredoxin-thioredoxin reductase
140	Os04g0460600	LOC_Os04g38720	2.1	NAC domain-containing protein 92
141	Os05g0429000	LOC_Os05g35440	2.1	Serine hydroxymethyltransferase 7 (SHMT)
142	Os06g0202700	LOC_Os06g10130	2.1	Unknown
143	Os08g0446800	LOC_Os08g34700	2.1	GLUTAMINE DUMPER 2
144	Os10g0576600	LOC_Os10g42610	2.1	Tetratricopeptide repeat (TPR) protein
145	Os01g0733001	LOC_Os01g53210	2.1	Putative metal transporter Nramp3
146	Os09g0522000	LOC_Os09g35010	2.1	Dehydration-responsive element-binding protein 1B (DREB1B)
147	Os02g0767400	LOC_Os02g52850	2.1	G-type lectin S-receptor-like serine/threonine-protein kinase
148	Os04g0648900	LOC_Os04g55520	2.1	Ethylene-responsive transcription factor ERF008
149	Os02g0324700	LOC_Os02g21920	2.1	Unknown
150	Os06g0107800	LOC_Os06g01860	2.0	B3 DNA binding domain-containing protein
151	Os02g0768300	LOC_Os02g52930	2.0	WALLS ARE THIN1 (WAT1)-related protein, drug/metabolite transporter (DMT) family protein
152	Os03g0710000	LOC_Os03g50210	2.0	Unknown
153	Os02g0605900	LOC_Os02g39330	2.0	Chitinase 6 (Cht6)
154	Os05g0450600	LOC_Os05g37800	2.0	Unknown, DUF740

155	Os10g0560000	LOC_Os10g41060	2.0	VAN3-binding protein, Auxin canalization, plant pleckstrin homology-like domain-containing protein
156	Os01g0667900	LOC_Os01g47760	2.0	Monothiol glutaredoxin-S5 (GRXS5)
157	Os01g0612900	LOC_Os01g42730	2.0	GDSL esterase/lipase
158	Os03g0247900	LOC_Os03g14370	2.0	ACT domain-containing protein (ACR8)
159	Os05g0192100	LOC_Os05g10370	-2.0	Haloacid dehalogenase (HAD)-like, acid phosphatase
160	Os10g0572300	LOC_Os10g42190	-2.1	Plant intracellular Ras-group-related LRR protein 5 (PIRL5)
161	Os01g0196300	LOC_Os01g09990	-2.1	Basic helix-loop-helix domain-containing protein (bHLH25)
162	Os07g0154100	LOC_Os07g05940	-2.7	9-cis-epoxycarotenoid dioxygenase (NCED)

Total RNA extracted from roots of nitrogen-starved 17-day-old rice seedlings (-N) or treated with 1.43 mM ammonium nitrate for 30 min was used for microarray analysis. Quantitative RT-PCR analysis was used to verify the expression of genes identified in the microarray data. The expression of genes listed here was up- or down-regulated by ammonium nitrate (1.43 mM, 30 min) for more than 2-fold in the qRT-PCR analysis.

Table S2. KEGG pathway enrichment analysis of genes (17 genes with KO) rapidly induced by ammonium nitrate (1.43 mM, 30 min) in rice roots

Pathway (ID)	Gene Identifier	Gene Description
Photosynthesis (ko00195)	Os01g0860601	Ferredoxin, root R-B1
	Os03g0784700	Ferredoxin--NADP reductase (FNR)
	Os05g0443500	Ferredoxin VI, chloroplast precursor
Nitrogen metabolism (ko00910)	Os02g0770800	Nitrate reductase
	Os01g0357100	Nitrite reductase
Sulfur metabolism (ko00920)	Os07g0509800	Adenylyl-sulfate reductase
	Os03g0196600	Serine O-acetyltransferase
Carbon metabolism (ko01200)	Os07g0406300	Glucose-6-phosphate 1-dehydrogenase
	Os11g0484500	6-Phosphogluconate dehydrogenase
	Os03g0196600	Serine O-acetyltransferase
	Os05g0429000	Glycine hydroxymethyltransferase
Pentose phosphate pathway (ko00030)	Os07g0406300	Glucose-6-phosphate 1-dehydrogenase
	Os11g0484500	6-Phosphogluconate dehydrogenase
Glutathione metabolism (ko00480)	Os07g0406300	Glucose-6-phosphate 1-dehydrogenase
	Os11g0484500	6-Phosphogluconate dehydrogenase
C5-branched dibasic acid metabolism (ko00660)	Os02g0608600	ACT domain containing protein ACR8, similar to acetolactate synthase I/III small subunit

Table S3. Gene ontology (GO) enrichment analysis of genes rapidly induced by ammonium nitrate (1.43 mM, 30 min) in rice roots

Locus ID		Gene Description
**Biological Process/regulation of transcription, DNA-dependent (GO:0006355)**		
Os02g0158000	LOC_Os02g06330	AP2/ERF transcription factor
Os06g0166400	LOC_Os06g07030	Ethylene-responsive transcription factor RAP2-9
Os01g0733200	LOC_Os01g53220	Heat stress transcription factor C-1b
Os01g0797600	LOC_Os01g58420	Ethylene-responsive transcription factor 8
Os01g0752500	LOC_Os01g54890	Pathogenesis-related genes transcriptional activator (PTI5); AP2/ERF transcription factor
Os04g0648900	LOC_Os04g55520	Ethylene-responsive transcription factor ERF008
Os02g0728001	LOC_Os02g49560	Basic leucine zipper 43-like (bZIP 43-like)
Os06g0107800	LOC_Os06g01860	B3 DNA binding domain-containing protein
Os09g0522200	LOC_Os09g35030	Dehydration-responsive element-binding protein 1A (DREB1A)
Os09g0522000	LOC_Os09g35010	Dehydration-responsive element-binding protein 1B (DREB1B)
Os01g0908200	LOC_Os01g68020	BTB/POZ and TAZ domain-containing protein 2
Os01g0292200	LOC_Os01g18800	CBL-interacting protein kinase 1 (CIPK1)
**Biological Process/protein amino acid phosphorylation (GO:0006468)**		
Os07g0584100	LOC_Os07g39520	Serine/threonine-protein kinase WNK5
Os02g0623600	LOC_Os02g41480	Wall-associated receptor kinase 5
Os01g0699500	LOC_Os01g50410	Mitogen-activated protein kinase kinasekinase NPKL3
Os01g0699400	LOC_Os01g50400	Mitogen-activated protein kinase kinasekinase NPKL2
Os01g0699600	LOC_Os01g50420	Mitogen-activated protein kinase kinasekinase NPKL4
Os09g0418000	LOC_Os09g25090	CBL-interacting protein kinase 16 (CIPK16)
Os12g0113500	LOC_Os12g02200	CBL-interacting protein kinase 14 (CIPK14)
Os01g0699100	LOC_Os01g50370	Mitogen-activated protein kinase kinase kinase NPK1
Os07g0538400	LOC_Os07g35390	Cysteine-rich receptor-like protein kinase 25
Os05g0545400	LOC_Os05g46760	Mitogen-activated protein kinase kinasekinase 2
Os11g0113700	LOC_Os11g02240	CBL-interacting protein kinase 15 (CIPK15)
**Biological Process/signal transduction (GO:0007165)**		
Os01g0292200	LOC_Os01g18800	CBL-interacting protein kinase 1 (CIPK1)
Os12g0113500	LOC_Os12g02200	CBL-interacting protein kinase 14 (CIPK14)
Os11g0113700	LOC_Os11g02240	CBL-interacting protein kinase 15 (CIPK15)
Os04g0450900	LOC_Os04g37790	Myosin heavy chain, SMC chromosome segregation protein
Os09g0418000	LOC_Os09g25090	CBL-interacting protein kinase 16 (CIPK16)
**Molecular Function/transcription factor activity (GO:0003700)**		
Os06g0166400	LOC_Os06g07030	Ethylene-responsive transcription factor RAP2-9
Os02g0158000	LOC_Os02g06330	AP2/ERF transcription factor

Os01g0733200	LOC_Os01g53220	Heat stress transcription factor C-1b
Os01g0797600	LOC_Os01g58420	Ethylene-responsive transcription factor 8
Os01g0752500	LOC_Os01g54890	Pathogenesis-related genes transcriptional activator (PTI5); AP2/ERF transcription factor
Os04g0648900	LOC_Os04g55520	Ethylene-responsive transcription factor ERF008
Os02g0728001	LOC_Os02g49560	Basic leucine zipper 43-like (bZIP 43-like)
Os09g0522200	LOC_Os09g35030	Dehydration-responsive element-binding protein 1A (DREB1A)
Os09g0522000	LOC_Os09g35010	Dehydration-responsive element-binding protein 1B (DREB1B)
**Cellular Component/nucleus (GO:0005634)**		
Os01g0780800	LOC_Os01g57240	ULTRAPETALA 1 (ULT1), trithorax group factor
Os02g0158000	LOC_Os02g06330	AP2/ERF transcription factor
Os06g0166400	LOC_Os06g07030	Ethylene-responsive transcription factor RAP2-9
Os01g0733200	LOC_Os01g53220	Heat stress transcription factor C-1b
Os01g0196300	LOC_Os01g09990	Helix-loop-helix domain-containing protein
Os01g0797600	LOC_Os01g58420	Ethylene-responsive transcription factor 8
Os01g0752500	LOC_Os01g54890	Pathogenesis-related genes transcriptional activator (PTI5); AP2/ERF transcription factor
Os04g0648900	LOC_Os04g55520	Ethylene-responsive transcription factor ERF008
Os02g0728001	LOC_Os02g49560	Basic leucine zipper 43-like (bZIP 43-like)
Os03g0230300	LOC_Os03g12820	RST and PARP domain-containing SRO (similar to RCD one) protein (SRO2)
Os09g0522200	LOC_Os09g35030	Dehydration-responsive element-binding protein 1A (DREB1A)
Os07g0685700	LOC_Os07g48630	ETHYLENE INSENSITIVE 3-like 1 protein, EIL2
Os09g0522000	LOC_Os09g35010	Dehydration-responsive element-binding protein 1B (DREB1B)
Os01g0908200	LOC_Os01g68020	BTB/POZ and TAZ domain-containing protein 2

Table S4. List of genes commonly induced by ammonium nitrate and glutamine

Locus ID		Fold change*		Gene description
		NH_4NO_3	Gln	
Os03g0445700	LOC_Os03g33090	15	2.7	LOB domain-containing protein 37 (LBD37)
Os09g0522200	LOC_Os09g35030	14.7	4	Dehydration-responsive element-binding protein 1A (DREB1A)
Os05g0114400	LOC_Os05g02390	14	5.4	Zinc finger protein, ZOS5-02
Os11g0184900	LOC_Os11g08210	11.5	4	NAC domain-containing protein, NAC5
Os07g0119300	LOC_Os07g02800	9.4	2.6	Unknown, MYB family protein
Os07g0589000	LOC_Os07g40000	8.5	2.3	LOB domain-containing protein 37 (LBD37)
Os01g0699100	LOC_Os01g50370	8.2	2.3	Mitogen-activated protein kinase kinase kinase (NPK1)
Os03g0823400	LOC_Os03g60840	5	3.5	Bowman-Birk type trypsin inhibitor
Os06g0633100	LOC_Os06g42660	4.9	3.6	Glutamine dumper 6
Os02g0205500	LOC_Os02g11070	3.1	2.2	3-Ketoacyl-CoA synthase 11
Os05g0194900	LOC_Os05g10650	2.9	2.4	ATP-dependent 6-phosphofructokinase 6
Os05g0402900	LOC_Os05g33400	2.9	2.4	Xylanase inhibitor I-like, basic 7S globulin
Os02g0687200	LOC_Os02g46210	2.3	2.6	Unknown, DUF581
Os08g0446800	LOC_Os08g34700	2.1	2	GLUTAMINE DUMPER 2

Genes induced by glutamine (2.5 mM, 30 min) were derived from Kan *et al.*[35]. Genes induced by ammonium nitrate (1.43 mM, 30 min) were from this study. *Fold change indicates the ratio of $+NH_4NO_3$ or +Gln/-N.

Table S5. List of genes commonly induced by ammonium nitrate and glutamate

Locus ID		Fold change		Gene description
		NH_4NO_3	Glu	
Os09g0484900	LOC_Os09g31130	15.7	2.3	Dicarboxylate transporter, solute carrier 13 (SLC13)
Os01g0915000	LOC_Os01g68650	14.4	2.7	Unknown, DUF506
Os11g0184900	LOC_Os11g08210	11.5	2	NAC domain-containing protein, NAC5
Os07g0119300	LOC_Os07g02800	9.4	2.1	Unknown, MYB family protein
Os07g0589000	LOC_Os07g40000	8.5	2	LOB domain-containing protein 37 (LBD37)
Os08g0492500	LOC_Os08g38460	5.3	2	Probable E3 ubiquitin-protein ligase XERICO
Os03g0823400	LOC_Os03g60840	5	4.7	Bowman-Birk type trypsin inhibitor
Os07g0583600	LOC_Os07g39470	3.8	2.1	Chitin-inducible gibberellin-responsive protein 2 (CIGR2), GRAS family protein
Os02g0205500	LOC_Os02g11070	3.1	2.6	3-Ketoacyl-CoA synthase 11
Os01g0705200	LOC_Os01g50910	3	3.7	Late embryogenesis abundant protein, group 3
Os05g0402900	LOC_Os05g33400	2.9	4.3	Xylanase inhibitor I-like, basic 7S globulin
Os08g0473900	LOC_Os08g36910	2.7	2.7	Alpha-amylase isozyme 3D (AMY3D)
Os07g0592600	LOC_Os07g40290	2.5	2.8	Indole-3-acetic acid-amido synthetase GH3.8
Os02g0687200	LOC_Os02g46210	2.3	3.1	Unknown, DUF581
Os03g0218400	LOC_Os03g11900	2.2	2.2	Sugar transport protein 13
Os02g0605900	LOC_Os02g39330	2	3.4	Chitinase 6 (Cht6)

Genes induced by glutamate (2.5 mM, 30 min) were derived from Kan *et al.*[36]. Genes induced by ammonium nitrate (1.43 mM, 30 min) were from this study. *Fold change indicates the ratio of $+NH_4NO_3$ or +Glu/-N.

Table S6. List of genes commonly induced by high and low concentrations of ammonium nitrate in rice roots

Locus ID		Fold change*	Gene description
1. Os03g0609500	LOC_Os03g41330	30.5	LOB domain-containing protein 38 (LBD38)
2. Os09g0522200	LOC_Os09g35030	14.7	Dehydration-responsive element-binding protein 1A (DREB1A)
3. Os11g0184900	LOC_Os11g08210	11.5	NAC domain-containing protein, NAC5
4. Os07g0119300	LOC_Os07g02800	9.4	MYB family protein
5. Os02g0595900	LOC_Os02g38230	5.2	High-affinity nitrate transporter 2.1 (NAR2.1)
6. Os03g0776400	LOC_Os03g56500	3.5	Unknown
7. Os03g0838900	LOC_Os03g62240	2.9	Unknown, mTERF domain-containing protein
8. Os06g0166400	LOC_Os06g07030	2.8	Ethylene-responsive transcription factor RAP2-9
9. Os05g0454500	LOC_Os05g38040	2.2	Unknown
10. Os04g0528800	LOC_Os04g44650	2.1	Ferredoxin-thioredoxin reductase
11. Os02g0770800	LOC_Os02g53130	65.8	Nitrate reductase [NAD(P)H]
12. Os01g0860601	LOC_Os01g64120	42.2	Ferredoxin, root R-B1
13. Os01g0631200	LOC_Os01g44050	32.3	Uroporphyrinogen-III C-methyltransferase
14. Os03g0684700	LOC_Os03g48030	25.8	Unknown, HPP family protein
15. Os01g0915000	LOC_Os01g68650	14.4	Unknown, DUF506
16. Os04g0640900	LOC_Os04g54830	13.4	Unknown
17. Os03g0233000	LOC_Os03g13050	10.4	Calcium uniporter protein 4, mitochondrial
18. Os03g0784700	LOC_Os03g57120	8.5	Ferredoxin--NADP reductase (FNR)
19. Os03g0764600	LOC_Os03g55590	8	MYB family protein
20. Os01g0357100	LOC_Os01g25484	8	Nitrite reductase
21. Os07g0406300	LOC_Os07g22350	5.1	Glucose-6-phosphate 1-dehydrogenase 2
22. Os11g0484500	LOC_Os11g29400	4.3	6-Phosphogluconate dehydrogenase 2 (6PGDH2)
23. Os03g0233900	LOC_Os03g13140	4.2	Non-symbiotic hemoglobin 1 (HB1)
24. Os01g0621900	LOC_Os01g43370	2.9	uORF-containing transcript
25. Os05g0443500	LOC_Os05g37140	2.9	Ferredoxin-6
26. Os02g0575700	LOC_Os02g36590	2.5	Loricrin
27. Os01g0747300	LOC_Os01g54340	2.2	Unknown, DUF506

Genes induced by high concentration ammonium nitrate (20 mM ammonium and 40 mM nitrate, 2h) were derived from Obertello *et al.*[40]. Genes induced by low concentration ammonium nitrate (1.43 mM, 30 min) were from this study.

*Fold change indicates the ratio of $+NH_4NO_3$/-N derived from this study.

Table S7. List of genes commonly induced by ammonium and ammonium nitrate in rice roots

Locus ID		Fold change*	Gene description
1. Os02g0770800	LOC_Os02g53130	65.8	Nitrate reductase [NAD(P)H]
2. Os01g0860601	LOC_Os01g64120	42.2	Ferredoxin, root R-B1
3. Os01g0631200	LOC_Os01g44050	32.3	Uroporphyrinogen-III C-methyltransferase
4. Os03g0684700	LOC_Os03g48030	25.8	Unknown, HPP family protein
5. Os01g0915000	LOC_Os01g68650	14.4	Unknown, DUF506
6. Os04g0640900	LOC_Os04g54830	13.4	Unknown
7. Os03g0233000	LOC_Os03g13050	10.4	Calcium uniporter protein 4, mitochondrial
8. Os03g0784700	LOC_Os03g57120	8.5	Ferredoxin--NADP reductase (FNR)
9. Os03g0764600	LOC_Os03g55590	8	MYB family protein
10. Os01g0357100	LOC_Os01g25484	8	Nitrite reductase
11. Os07g0406300	LOC_Os07g22350	5.1	Glucose-6-phosphate 1-dehydrogenase 2
12. Os11g0484500	LOC_Os11g29400	4.3	6-Phosphogluconate dehydrogenase 2 (6PGDH2)
13. Os03g0233900	LOC_Os03g13140	4.2	Non-symbiotic hemoglobin 1 (HB1)
14. Os01g0621900	LOC_Os01g43370	2.9	uORF-containing transcript
15. Os05g0443500	LOC_Os05g37140	2.9	Ferredoxin-6
16. Os02g0575700	LOC_Os02g36590	2.5	Loricrin
17. Os01g0747300	LOC_Os01g54340	2.2	Unknown, DUF506
18. Os01g0606900	LOC_Os01g42190	20.5	DnaJ homolog subfamily B member 7
19. Os03g0445700	LOC_Os03g33090	15	LOB domain-containing protein 37 (LBD37)
20. Os05g0525900	LOC_Os05g45020	14.7	Zinc finger CCCH domain-containing protein 37
21. Os05g0114400	LOC_Os05g02390	14	Zinc finger protein, ZOS5-02
22. Os08g0452900	LOC_Os08g35160	9.4	DnaJ/heat shock protein 40 (Hsp40)
23. Os07g0589000	LOC_Os07g40000	8.5	LOB domain-containing protein 37 (LBD37)
24. Os01g0699100	LOC_Os01g50370	8.2	Mitogen-activated protein kinase kinase kinase (NPK1)
25. Os03g0243100	LOC_Os03g13950	6.5	Actin-depolymerizing factor 5 (ADF5)
26. Os09g0502500	LOC_Os09g32570	6.5	Quinone-oxidoreductase homolog
27. Os01g0130900	LOC_Os01g03980	6.2	Zinc finger protein ZIC 2
28. Os03g0114400	LOC_Os03g02330	5.1	AAA-ATPase
29. Os06g0633100	LOC_Os06g42660	4.9	Glutamine dumper 6
30. Os03g0807900	LOC_Os03g59320	3.9	Unknown
31. Os05g0194900	LOC_Os05g10650	2.9	ATP-dependent 6-phosphofructokinase 6
32. Os01g0212700	LOC_Os01g11460	2.8	RING-H2 finger protein ATL39
33. Os08g0473900	LOC_Os08g36910	2.7	Alpha-amylase isozyme 3D (AMY3D)
34. Os06g0208700	LOC_Os06g10650	2.5	Tyrosine phosphatase family protein
35. Os04g0583200	LOC_Os04g49370	2.4	Unknown
36. Os02g0748300	LOC_Os02g51350	2.2	F-box/kelch-repeat protein
37. Os02g0324700	LOC_Os02g21920	2.1	Unknown

38. Os02g0768300	LOC_Os02g52930	2	WALLS ARE THIN1 (WAT1)-related protein, drug/metabolite transporter (DMT) family protein

Genes induced by ammonium (1 mM, 3h) were derived from Chandran *et al.*[25]. Genes induced by ammonium nitrate (1.43 mM, 30 min) were from this study. *Fold change indicates the ratio of +NH_4NO_3/-N derived from this study.

Table S8. List of genes commonly induced by ammonium and ammonium nitrate in rice roots

Locus ID		Fold change*	Gene description
1. Os02g0770800	LOC_Os02g53130	65.8	Nitrate reductase [NAD(P)H]
2. Os01g0860601	LOC_Os01g64120	42.2	Ferredoxin, root R-B1
3. Os01g0631200	LOC_Os01g44050	32.3	Uroporphyrinogen-III C-methyltransferase
4. Os03g0684700	LOC_Os03g48030	25.8	Unknown, HPP family protein
5. Os01g0915000	LOC_Os01g68650	14.4	Unknown, DUF506
6. Os04g0640900	LOC_Os04g54830	13.4	Unknown
7. Os03g0233000	LOC_Os03g13050	10.4	Calcium uniporter protein 4, mitochondrial
8. Os03g0784700	LOC_Os03g57120	8.5	Ferredoxin--NADP reductase (FNR)
9. Os03g0764600	LOC_Os03g55590	8	MYB family protein
10. Os01g0357100	LOC_Os01g25484	8	Nitrite reductase
11. Os07g0406300	LOC_Os07g22350	5.1	Glucose-6-phosphate 1-dehydrogenase 2
12. Os11g0484500	LOC_Os11g29400	4.3	6-Phosphogluconate dehydrogenase 2 (6PGDH2)
13. Os03g0233900	LOC_Os03g13140	4.2	Non-symbiotic hemoglobin 1 (HB1)
14. Os01g0621900	LOC_Os01g43370	2.9	uORF-containing transcript
15. Os05g0443500	LOC_Os05g37140	2.9	Ferredoxin-6
16. Os02g0575700	LOC_Os02g36590	2.5	Loricrin
17. Os01g0747300	LOC_Os01g54340	2.2	Unknown, DUF506

The 158 genes identified in this study were compared with the genes reported by Chandran *et al.*[25] and Obertello *et al.*[40]. *Fold change indicates the ratio of +NH_4NO_3/-N derived from this study.

Table S9. Sequences of primers used for quantitative RT-PCR analysis

No.	Locus ID		Primer sequence 5'→3'
1	Os02g0770800	LOC_Os02g53130	CGGAGAAGCTCATTTGGAATCT/CACCCTGAACCAGCAGTTGTT
2	Os01g0860601	LOC_Os01g64120	CTCCTGTAGCGACATGCTTCGT/CCGTGCGCTATAATTGGTGATC
3	Os01g0631200	LOC_Os01g44050	TCCCAAGTTGATGAAGCATGG/CAACTGCAACAGCCGGTGTAT
4	Os03g0609500	LOC_Os03g41330	TTTTTGGTCATGTGCAACTT/ATAGTCTGCAGCTAGCATTG
5	Os03g0684700	LOC_Os03g48030	GCAGTCTGCGATGTGCTTTTC/TGTATTTCTTGGCAGCAGGTGA
6	Os01g0606900	LOC_Os01g42190	GCGTACCAAGTGTTGTCTGACG/TACATGCCTGAGTCGTAGAGCG
7	Os02g0728001	LOC_Os02g49560	TGCCAGCTGAGAGATGAACAAG/GGTCTTCTCAAGCTGCTTCTGC
8	Os09g0484900	LOC_Os09g31130	GGAACGCCCCTGAAAATTGT/TGGCAGTAGGATGGTGAGAGCT
9	Os03g0445700	LOC_Os03g33090	GTTGTTCCAGTCGCTGCTGTAC/TGCACCGGATTGATGGTTC
10	Os05g0525900	LOC_Os05g45020	CATCGATGAATGCGACGC/ATTGCGAACATCAACTCTTCCA
11	Os09g0522200	LOC_Os09g35030	ACATGGGCTGGGACCTGTAC/TCCATGAGCATCCCCTGC
12	Os01g0915000	LOC_Os01g68650	TCGGCGATGAAACATCATAA/GAAAGGAACTCTCGCTCTAA
13	Os05g0114400	LOC_Os05g02390	AAGAGGTTTGCATTCGAGGACA/CATGAGCATCAGCACGCG
14	Os04g0640900	LOC_Os04g54830	AGCCAACATTTTGCCATAGCC/CCTCTCGAAATGCCCAACATC
15	Os11g0184900	LOC_Os11g08210	GATCAAGAAGGCGCTCGTCTT/CGTGCATGATCCAGTTGGTCT
16	Os03g0233000	LOC_Os03g13050	ATGATAGCGAAAGCGATCGG/TCCCGTTTCTTCCGCACTT
17	Os07g0119300	LOC_Os07g02800	CGAGAGTGCCAAGCTCAACAA/GGAATGTAACAGAGCCCTTGCA
18	Os08g0452900	LOC_Os08g35160	CATGAACAAAACCGGCCCTAC/AACAGCTCCTCGAACTGCCTCT
19	Os07g0589000	LOC_Os07g40000	TTTTTGGCCGTGTAAAAGTC/TGATGCTAACAAAGTAGGGG
20	Os03g0784700	LOC_Os03g57120	AGCTACTGGCACTGGTGTTGCT/TGAACATACGGCGTAGGTAGCC
21	Os01g0699100	LOC_Os01g50370	GATATCCAAGCCGCTGATCGT/AGCGCAGACTTTATTCCGACG
22	Os03g0764600	LOC_Os03g55590	CCCCCAAGTTGCTACTCCAAA/GGTGAGCCCATCAACCTTCAT
23	Os01g0357100	LOC_Os01g25484	TCGACAAGAAATGGCAGAGGAG/ACGTAGGACATCCCTTCCTGCT
24	Os04g0480200	LOC_Os04g40410	TGTCAGTTCAAGATCGCCCAG/TGCCACCGTGTACTCGAACTT
25	Os03g0243100	LOC_Os03g13950	ATCTTCTTCATCGCCTGGTCAC/TCGCGTACAGAATCTTGGCTC
26	Os09g0502500	LOC_Os09g32570	AGGCGAGGTGCTGATCAAGAT/TTCCAGTCGACCTGGTTGATG
27	Os01g0130900	LOC_Os01g03980	ACGCCGAGATGGCCTTC/CGTAGATTTGGTGGATCCTCCA
28	Os05g0360400	LOC_Os05g29710	GGTTTATGCACACCATTGGCA/CTGACAACACAAGCAGCAAACA
29	Os03g0126700	LOC_Os03g03500	TCTTGCATTGCATCTGATCT/GAGAAAATGTACGTAGGGCT
30	Os07g0530600	LOC_Os07g34640	TGACGCATCATCGACGGT/CGCTGTCGACTGCTACTA
31	Os11g0113700/ Os12g0113500	LOC_Os11g02240/ LOC_Os12g02200	TTTCGGAGCAGATCAGACGTG/TTATGAGCCACCAACCGCAT
32	Os08g0492500	LOC_Os08g38460	ATCTCGAGCATGCCGGC/TGTTGTACAGCAGGTACGCCAC
33	Os03g0148400	LOC_Os03g05460	TGGATTGCGAAATTCAGCCT/TTGCTCTGACTGAACCATGGC
34	Os02g0595900	LOC_Os02g38230	AAGGCCTCCGACGACCTG/CGGTGACCTTGAACTGGCA
35	Os01g0646000	LOC_Os01g45840	TTTCAAGCCATGCCTGCAA/GCCATCGATCAACGTATTCCAT
36	Os03g0114400	LOC_Os03g02330	ATCAAGATCAGGAGCCGGAA/GTGCAAGGGCATGAACCC
37	Os07g0406300	LOC_Os07g22350	TGCCATTGAAGGAGAGCGAA/TGCGGCATCCAACTCATCA
38	Os05g0455300	LOC_Os05g38130	TGACGCATCATCGACGGT/CGCTGTCGACTGCTACTA

39	Os02g0535901	LOC_Os02g33240	CCAAGAGGAGGTACGACGAGTT/ACTCCTTCCTGCCCCTCTTC
40	Os03g0823400	LOC_Os03g60840	CCACCACTCCCATCTCCATG/TGCTTCTCCTCCTCTCTCGC
41	Os01g0752500	LOC_Os01g54890	GTCGTCGTACCTCCCGCTC/AAGAGGACCATGTCCAGCGA
42	Os06g0633100	LOC_Os06g42660	GGACCAATTTTGTTCGTGAG/GATGATTACAGGGGTGGAAA
43	Os05g0443700	LOC_Os05g37150	AAGCCTCAATGTTCATGCGG/CAAACCACGCATACTGCTACCA
44	Os01g0733200	LOC_Os01g53220	TCCTACTTCAAGCACCGCAACT/GGAATCCGTAGGTGTTGAGCTG
45	Os06g0566300	LOC_Os06g37010	CTGCAACCCAAGGCTTCAAGT/TGGCCCCAAGAAACAGAGCTA
46	Os11g0484500	LOC_Os11g29400	GCACACACCTATGAGCGCATT/CCACTCTGTGTGGAACGAACCT
47	Os03g0759000	LOC_Os03g55180	CATTCAGGCACAGAAGGCAGA/GCCTCATGCAGCACAAGACAT
48	Os03g0233900	LOC_Os03g13140	TCGAGAAGAACCCCAAGCTCA/TCATGACGAAGACGGACATGG
49	Os11g0100300	LOC_Os11g01030	GCAGCAAGATCAAGGAGTAGCA/TCTCCCATGCAGAACCCAAT
50	Os01g0780800	LOC_Os01g57240	TTCACTGTACGGAATGTGGCAA/ACTCTTCTTTAGACCGGAGCCG
51	Os01g0645900	LOC_Os01g45830	TCGTGCTGTACCTGTTGCTACG/CGAGCAGTCCTCTGATATCCCA
52	Os05g0497800	None	AGGGGAGGAAAAGGAGGAGGA/AAGTCCTCCACGCGCCACGGCCA
53	Os03g0807900	LOC_Os03g59320	AGTGTTCCTTCGTGCTCTTGGA/TCATCACTCGCTCTGCAAGCT
54	Os07g0583600	LOC_Os07g39470	AGAGGATCAACATGGAGCAGCA/ACGCGATGAGGTTGACGATCT
55	Os02g0713700	LOC_Os02g48320	GTGGCATGTCGCTCTACAACCT/GCTCCAACCTCCGAAGTTGTCT
56	Os01g0948200	LOC_Os01g71970	CATGGCGCTCACGAAAGAA/TTGAACTGGAACGGCACGT
57	Os01g0699600	LOC_Os01g50420	TGAAGTTTCGGAAACACCGG/CACCAAAGACCGAAATCTGCA
58	Os02g0623600	LOC_Os02g41480	TGACAAGA CGATAGGCAACGC/GGACTTGTTATCCCCGCATGT
59	Os02g0775600	LOC_Os02g53530	GGAGGTGGCATGCAAGGA/GACGAGGACAGCAAGCTCATC
60	Os09g0418000	LOC_Os09g25090	TCGCTGGGTTACAGCGC/CTCCAACCTCAGCTTCCACC
61	Os02g0693000	LOC_Os02g46620	AATTAACTAGACGGGGGAGA/ATCATAAGATTAGCACGCGA
62	Os03g0776400	LOC_Os03g56500	ACGAGCACGGAATCTGGGA/TGAGATGGTTGCAGTAT
63	Os05g0179100	LOC_Os05g08620	GAGTCGACTTTGATAAGGGG/TATCCTCAGCATCCTCACAT
64	Os07g0509800	LOC_Os07g32570	ACGTACCAGCTGTTCGACAAG/TCGTAGAAGGAGAAGAG
65	Os01g0314800	LOC_Os01g21250	TTGCATCGAGCTGTCTCAGC/CCACCGAGTAACCCCTCCTT
66	Os02g0205500	LOC_Os02g11070	GTTGGGTTTTGTGGTACTTG/TCCTGTTCCTACCCTAACAA
67	Os04g0475600	LOC_Os04g39980	TGGCCGTGCCAGTTCC/AGCCCACGGAGTCCTGC
68	Os10g0203000	LOC_Os10g13550	CTTCTGCCAGGCACTTTGTCA/TTTAGGCGATCAGGATGGACC
69	Os01g0525800	None	GCTTTTGTGCAACGTCGAAA/CAATCCAAACGATCCCACTACA
70	Os03g0196600	LOC_Os03g10050	GCCAAGATGGTCCACTGCTT/GGGCCTGCAGTGCCAG
71	Os02g0158000	LOC_Os02g06330	GCGCAACGGCGGCAGCAA/GCAGAGCACGGAGGAAGA
72	Os02g0533700	LOC_Os02g33060	TGATGGCGAGTATGACCCGT/GATCCTGAGCCACAGCGACT
73	Os01g0705200	LOC_Os01g50910	GGCGATAACAAGAACAACGCC/AGTCACTCCCAGTTCCAGGCTT
74	Os01g0266400	LOC_Os01g16140	GAGAGCACCAGCGTAATGGC/GAGGCTGTGTCCGCTCATGTA
75	Os01g0823900	LOC_Os01g60860	CCGTCGTGGAGAAATTAGTG/GCCAATGCGTCCTTCTTTGT
76	Os09g0246500	LOC_Os09g07150	GCCATGCGCGTCATTTTT/GGTAGTCGCCGTCGGAAA
77	Os01g0699400	LOC_Os01g50400	TCGAAGCGTTGGAAACGATC/ATCAACACACTCTGCATCGCC
78	Os07g0584100	LOC_Os07g39520	TGCAGCAACGTCTTCATCAAC/GTCCCCGATCTTGACCTGG
79	Os05g0402900	LOC_Os05g33400	AATACTTCGTCGGCTTGACGC/TTGTTGATGGCAAGCAGCG

80	Os05g0194900	LOC_Os05g10650	CAAGCAGTACTTCGTGAACGCC/TGTGCACCACGATCTTCTTCG
81	Os01g0621900	LOC_Os01g43370	AGTCATGATGGTTGTTCCCGG/TGATGCACAAGCCAGATTTGC
82	Os03g0838900	LOC_Os03g62240	GAGGACATTCTCCGCTGATCAA/TGTTGTCACCAGCATCGACAA
83	Os03g0275900	LOC_Os03g16824	CCAAAAGTGGCACAGACAGAGG/CGCTCAAGAAGCTGGATTGCT
84	Os05g0443500	LOC_Os05g37140	GCGGTGCACAAGGTAAAGCTT/GCTTCAAACTCGTGCTCGACA
85	Os01g0700100	LOC_Os01g50460	AGGCAACATCTTTGCGCTTG/TCTTAAACGTCGTCACTGGCG
86	Os04g0523100	LOC_Os04g44210	CGCAATGAAGTGTCAGTGGCT/TTCCGCCGCCTTGAATTT
87	Os03g0175200	LOC_Os03g07890	CATTTGGTTTCTCGATCTGC/TTTGCATCCTAAAACCCCTT
88	Os04g0567800	LOC_Os04g47990	AGAAGGAGAAGGCGCTCAACTG/GCAGAACTTGGTGTTCGTCGA
89	Os03g0292600	None	TAGGGCGAGCTGAGCCGCCTCAAGAT/ATGGCGTGCCTCGAGAGCGAGATAA
90	Os01g0212700	LOC_Os01g11460	TTCTCCATCGGCATCCTCTC/GGTGTACCAGATGGCGAGGT
91	Os06g0166400	LOC_Os06g07030	GAGGAGCCCACAAGTATATC/TACTTGCTAACCACCACTTC
92	Os05g0545400	LOC_Os05g46760	AGAATTCGAGCTTCCCCACATT/TGTGCTGAGCATTGGCTTCTC
93	Os07g0593000	LOC_Os07g40300	TCCATGGAGGCCAGATGAGTAG/CCATGTTCAACCGCAAAAGG
94	Os04g0450900	LOC_Os04g37790	AGCCACTACGACAGCATGGATC/TTGCGAAGTATCGGCCTCTTC
95	Os08g0473900	LOC_Os08g36910	CACCAGCAGCTTGTGTTTGCT/TGCTTGACCCGAGTTACAGGTC
96	Os01g0292200	LOC_Os01g18800	CCTTCCCTTTGATGACCGAAA/GGATTTGAGTGTCCCCCTTGA
97	Os07g0672600	LOC_Os07g47540	TGCAGCGACAATGTCTACCTCA/ATGTTCAGGACGATCTTGCCG
98	Os08g0207500	LOC_Os08g10630	TCATGGCGACTTTCTTCTCCC/AGCTGGATGAGATTGCGATCC
99	Os09g0103400	LOC_Os09g01610	GCAATTCAAGGAGAAGATTGCG/GAAACCACCTTCCCAAGGAAAA
100	Os02g0530300	LOC_Os02g32840	TTCCTCGCCATCCTCGTC/GCAGTGGAGGAAGGCAAGAAC
101	Os05g0560200	LOC_Os05g48650	TGGAGATCGGCTTCCG/CTAGTCGCTGCGGGC
102	Os09g0400700	LOC_Os09g23590	TTCGAGGTCAGCTCCAATGGA/ACCGCTGATCTTCTTGCTCCCT
103	Os03g0242300	LOC_Os03g13870	CAACGTTCTTCTGGATCGCC/CTCCTCGGGAACTCGAAGC
104	Os03g0297600	LOC_Os03g18600	GTTCGTCGACACCATCGTCA/GCGGTCTTGGCGAGAGACT
105	Os06g0208700	LOC_Os06g10650	GGAACCATTCGTCAACATCCC/ACTTTGAGCGCCTCTCGAATTT
106	Os02g0575700	LOC_Os02g36590	CGACGATTCAGCATCTGGAAGA/GCGAAGGAGATGATGTTCCCA
107	Os07g0592600	LOC_Os07g40290	TCATGCCGGTCATGAACTTGT/CGAACAGGAAGTAGAGCCCCTT
108	Os03g0230300	LOC_Os03g12820	ACCAGTTCATCCGGTTCTTGC/CCACGGTGGTCAACACATTGT
109	Os02g0608600	LOC_Os02g39570	TGTCAATGATTGCCCTGGTGT/CGAGCAAAGACCCCTGTAACAA
110	Os04g0583200	LOC_Os04g49370	ATTTGCTTAGGAGATTTGC/GCTTGACATGAGTGCGTACA
111	Os01g0797600	LOC_Os01g58420	ACCGTATGATGAAGCTTGCA/TCGGAGTCACTTTGTGCAA
112	Os01g0908200	LOC_Os01g68020	GCATGCAGTTCAGGAGAAAGGA/TTCACCAGAAGCCCCCACTTA
113	Os02g0687200	LOC_Os02g46210	CCAGGAACTGCGACATCTTCAT/TAACCCTCACTGCTGCTTCGTC
114	Os.22947.1.S1_at	LOC_Os04g06080	TCCTGAGGAACAGGCAAGTC/TCGCTGGTTTCGAGGATACG
115	Os04g0649500	LOC_Os04g55600	GCAGGCGTTCTTGCAGTACTC/AGCACGAGGTTGAACGGC
116	Os02g0771100	LOC_Os02g53140	AAATTGCGAAGACAGCATCACC/CCTGTTGCAGTGCATATCGAAA
117	Os01g0504100	LOC_Os01g31940	TCTGCCTCTGGGCCTTCTC/CCCCTTTCTTGTACTCCCCATAG
118	Os01g0787400	LOC_Os01g57740	GACGCCAATCCTTCTACCACAG/ACCACCTGCTTCTCATACACGG
119	Os05g0181800	None	CGGAGTAGGAGGAAATGGTCAC/GCCGGTTTCTACCTACTGTTCG
120	Os07g0602900	LOC_Os07g41160	AGGAAGAGCATGATGGAGGACA/TTGTACAGGAACCCGTCGATCT

121	Os04g0649600	LOC_Os04g55610	GGGCTGGTTGTAAGTTGTAA/TGGCCGTATTTCACTTGTAT
122	Os10g0546400	LOC_Os10g39920	AGCTAGAGTAGTTCGGAGTT/AGTGCAAGTACAGCAGTAAA
123	Os02g0689200	LOC_Os02g46380	CCCCTCTGATTTTCAATTCCGT/CCAAGGAGTGAAGCAGAAAACG
124	Os01g0747300	LOC_Os01g54340	TGCATGAAGGAGAACAACATGC/GCATGTACTTGTGCTTCCTCCA
125	Os02g0160200	LOC_Os02g06520	GCACTGCCCCATTTTCTCAA/GCGATTGCTGAAGCTCTTTCA
126	Os05g0454500	LOC_Os05g38040	ATCGGAGTCGCCATATTTGG/AAACTCCCGAGCACCGTAATG
127	Os02g0748300	LOC_Os02g51350	GGAACCATGAGTGTGAGCAGCT/CCGAAAAACCCTGATCATTGC
128	Os01g0699500	LOC_Os01g50410	AGAAGCGATCGAATCCTCCATT/TGCCAAGATGTACGTGCAGGT
129	Os07g0538400	LOC_Os07g35390	TTTTCGGTCGAGATCAGACCC/TATGTGCCGATGACATTCTTGG
130	Os10g0541200	LOC_Os10g39540	CAATTGCGGATTCCCATTCTC/TGCTGTCAATGCTGTAGTCCCA
131	Os03g0218400	LOC_Os03g11900	ACATCCCCATCGAGGAGATGA/AGAACCAGTGCTGCTTCCACAC
132	Os04g0405300	LOC_Os04g33240	CGTTCATGAGTCTGCAAGAAAGTT/CCGGTGATCAGCGCCA
133	Os04g0671200	LOC_Os04g57550	AAAATGCTACCTGACGCCACTG/ACCCCAGCGTGAAACCAAAT
134	Os02g0668500	LOC_Os02g44820	CGGGCTGTACGAGAAGCTG/TCCCACAGGTACTCGAGCCT
135	Os01g0101700	LOC_Os01g01160	AGGAGGAGCATGACGAAGGA/CCGAGCTCCCCAGGAGAG
136	Os07g0685700	LOC_Os07g48630	GCTCTGAGCAACAACAAGTTCC/GTCGATGGCCTCTTCCTTCAT
137	Os07g0225300	LOC_Os07g12340	TGTGCCGGCTGTACAACAA/TTCCTGCTCTGCATCTTCTCC
138	Os01g0812200	LOC_Os01g59690	GCCTGCAACATGAAGCTGAAAT/TCATGCCACCAGGTAGCTCAA
139	Os04g0528800	LOC_Os04g44650	CAATCTCCCCTTCAAGGTGGA/AAGAACCGGACCGGTTTGT
140	Os04g0460600	LOC_Os04g38720	GGACAAGGACATCTTCAGACGG/AACGAGCGTCTTCTTCATGCC
141	Os05g0429000	LOC_Os05g35440	AAAAATGCCCAAGCTTTGGC/CCAGTCTGCATTTCCTCCTGAG
142	Os06g0202700	LOC_Os06g10130	CGCAGGTCGTTTACCTCATCCT/AGGAGCCAAGAAGACGTGAAGC
143	Os08g0446800	LOC_Os08g34700	CGACGAGAAGAAGAAGGTGCAG/TGCTCACCATTCTCACCATCCT
144	Os10g0576600	LOC_Os10g42610	ACTGCTACGTGCTTGGATCGT/CCTCTTCCGCGTCCCAC
145	Os01g0733001	LOC_Os01g53210	GAAGGATGGGGCTAAGAGT/AATGTAGCCAGTCTCTCCAC
146	Os09g0522000	LOC_Os09g35010	GTCTCCGGCGGAGACCTT/TTGTTGTCTTCTTCTTCGTCGC
147	Os02g0767400	LOC_Os02g52850	GCAACGACATTGTGATCCCTG/TGGTCATGTCCTCGATCTCCTC
148	Os04g0648900	LOC_Os04g55520	CAAGAACCCGGATCTCAAC/CGGCGTCGGAGTTCT
149	Os02g0324700	LOC_Os02g21920	TTCCTGCTTTACACCGCCAA/AGTAGATCTTCTCGTCGGCGCT
150	Os06g0107800	LOC_Os06g01860	GGCGCTTCCGCTACTCCTA/TGGTGATGACGTAGCTCTGGC
151	Os02g0768300	LOC_Os02g52930	TTCAACCCGCTCTGCATGA/TCCTTGAGAATGATGGAGCCC
152	Os03g0710000	LOC_Os03g50210	AAATCTGGCAGGATCAGACGTG/CGGACCACTTCATCATCCTCAG
153	Os02g0605900	LOC_Os02g39330	ACGCCAACCACGAGACCATAA/GTCCGTCGATCTCCTCGATGTA
154	Os05g0450600	LOC_Os05g37800	TGGAAACGGAAGCCAATGGT/ATGTGGTCCTTCATGGCCTTG
155	Os10g0560000	LOC_Os10g41060	TTCCCAGCCATCTGCAACAT/GGTGGCTGCTGATTGAATTCC
156	Os01g0667900	LOC_Os01g47760	GCGTACGTGATGATGAGTAT/AAATGTCACTGTCCCTCTTC
157	Os01g0612900	LOC_Os01g42730	CTTCACGGAGGCAACCTACAAG/GCATATGACCCTAGCACCCCTT
158	Os03g0247900	LOC_Os03g14370	ACGGACTTGGATTACGTGGTGT/ACGCCGTATGTAGAACTCCTGC
159	Os05g0192100	LOC_Os05g10370	CAAGGAGATCTGGGTGTTC/GTAGTAGGGGAGGTTGGAG
160	Os10g0572300	LOC_Os10g42190	GGCAAATTGCAAGATGTTACCG/TGCCATGATCCTATTCTCGGAA
161	Os01g0196300	LOC_Os01g09990	GCGATGAGACGAAGCGAAAG/CGCTCGTCGTCACCTCG

162	Os07g0154100	LOC_Os07g05940	ACTTCGCCGTCACCGAGAACTA/GCACCATCTCCTGGAGCTTGAA
163	UBC3<br>Os02g0634800	LOC_Os02g42314	AAGGCATGGCTGATTCATGT/CAGATGTTCACATGGTGACAGTA
164	UBQ10<br>Os02g0161900	LOC_Os02g06640	TGGTCAGTAATCAGCCAGTTTGG/GCACCACAAATACTTGACGAACAG
